# Actin-capping protein regulates actomyosin contractility to maintain germline architecture in *C. elegans*

**DOI:** 10.1242/dev.201099

**Published:** 2023-03-23

**Authors:** Shinjini Ray, Priti Agarwal, Anat Nitzan, François Nédélec, Ronen Zaidel-Bar

**Affiliations:** ^1^ Department of Cell and Developmental Biology, Faculty of Medicine, Tel Aviv University, 6997801 Tel Aviv, Israel; ^2^ Graduate Program, Mechanobiology Institute, National University of Singapore, 117411, Singapore; ^3^ Sainsbury Laboratory, University of Cambridge, 47 Bateman Street, Cambridge CB2 1LR, UK

**Keywords:** Actin dynamics, Capping protein, Cytoskeleton, Actomyosin, Contractility, *Caenorhabditis elegans*, Syncytial germline, Formins, Arp2/3 complex, Rachis, Morphogenesis, Cytosim, Fertility

## Abstract

Actin dynamics play an important role in tissue morphogenesis, yet the control of actin filament growth takes place at the molecular level. A challenge in the field is to link the molecular function of actin regulators with their physiological function. Here, we report an *in vivo* role of the actin-capping protein CAP-1 in the *Caenorhabditis elegans* germline. We show that CAP-1 is associated with actomyosin structures in the cortex and rachis, and its depletion or overexpression led to severe structural defects in the syncytial germline and oocytes. A 60% reduction in the level of CAP-1 caused a twofold increase in F-actin and non-muscle myosin II activity, and laser incision experiments revealed an increase in rachis contractility. Cytosim simulations pointed to increased myosin as the main driver of increased contractility following loss of actin-capping protein. Double depletion of CAP-1 and myosin or Rho kinase demonstrated that the rachis architecture defects associated with CAP-1 depletion require contractility of the rachis actomyosin corset. Thus, we uncovered a physiological role for actin-capping protein in regulating actomyosin contractility to maintain reproductive tissue architecture.

## Teaser

**Summary:** Genetic and biophysical manipulations, as well as *in silico* simulations, reveal a role for the *C. elegans* actin-capping protein CAP-1 in regulation of actomyosin contractility to maintain reproductive tissue architecture and fertility.

## INTRODUCTION

Diverse actin structures support the shape and power the movement of biological entities from the subcellular to the tissue level. Their functional diversity, manifested by different actin network architectures and dynamics, is regulated by their association with a variety of actin-binding proteins in a context-dependent manner (Pollard, 2016; Winder and Ayscough, 2005). The barbed end of the actin filament is a hotspot for regulation, whereby interactions between multiple proteins determines whether it will elongate or remain stable (Shekhar et al., 2016). Capping protein (CP, also known as CAPZ and β-actinin) is one of the major determinants of barbed end dynamics (Edwards et al., 2014). CP binding at the barbed end of the actin filament prevents addition or loss of actin subunits (Casella et al., 1986). It functions as a heterodimer composed of two structurally similar α and β subunits (Haus et al., 1991; Yamashita et al., 2003). Cryo-electron microscopy particle imaging revealed the structure of CP attached to the barbed end and suggested a two-step model for its binding, mediated by two tentacles (Narita et al., 2006). Several CP binding partners, including V1/myotrophin, CARMIL, CapZIP and twinfilin have been postulated to prevent CP from capping F-actin (Edwards et al., 2014; Eyers et al., 2005; Hakala et al., 2021; Johnston et al., 2018; Taoka et al., 2003; Yang et al., 2005).

CP is conserved throughout the eukaryotes (Cooper and Sept, 2008). In yeast, deletion of CP leads to abnormal actin distribution, including loss of actin patches and cables, and affects cell shape (Amatruda et al., 1990; Nakano and Mabuchi, 2006). In animals, CP has a well-established role in muscle cells, where it organizes F-actin in the Z-line of sarcomeres (Casella et al., 1987; Schafer et al., 1993; 1995). Additionally, CP was found to regulate endocytosis and endosomal trafficking by controlling F-actin density around endocytic sites and endosomes, respectively (Lamb et al., 2021; Wang et al., 2021). The involvement of CP in other cellular processes, such as spindle migration, polar body extrusion, abscission, and autophagosome formation, has been attributed to its effect on the cytoplasmic actin mesh density (Jo et al., 2015; Mi et al., 2015; Terry et al., 2018).

Recently, knockout of CP in cultured fibroblasts revealed that CP can also function to restrain actomyosin contractility and conditional knockout of CP in mouse hepatocytes led to hyperproliferation downstream of YAP activation, presumably due to increased contractility (Pocaterra et al., 2019). This study raised the possibility that CP functions *in vivo* to tune the mechanical properties of cells and tissues. However, the molecular basis of this phenomenon and whether it is unique to fibroblasts or a general function of CP remain unknown.

Force generated by actomyosin machinery is essential for cell and tissue morphogenesis as well as the physiological function of many tissues (Agarwal and Zaidel-Bar, 2019). One example is the syncytial germline of *Caenorhabditis elegans*, where inward contraction of a corset-like actomyosin structure lining the rachis balances the outward pulling force of germ cell membrane tension (Priti et al., 2018). In a large-scale RNAi screen that examined gonad architecture, depletion of CAP-1, the alpha subunit of CP, was found to lead to severe multinucleation with irregular cell size and severe vesiculation (Green et al., 2011). Also, *C. elegans* CAP-1 and CAP-2, the beta subunit of CP, were previously expressed and purified from yeast and shown to function similarly to chicken skeletal muscle CP in actin polymerization assays (Waddle et al., 1993). However, a detailed characterization of CAP-1 function in *C. elegans* and the molecular mechanisms that led to the germline defects observed in its absence are still missing. Here, we carried out a comprehensive investigation of the tissue and subcellular localization of CAP-1 in *C. elegans*, with an emphasis on the germline. We measured and simulated the effect of CAP-1 on F-actin and myosin levels and uncovered a role for CP in maintaining a balanced level of actomyosin contractility in the syncytial germline, which is required for the proper structure and function of the reproduction organ.

## RESULTS

### CAP-1 is widely expressed and is a component of the actomyosin corset in the syncytial germline

**Fig. 1.**
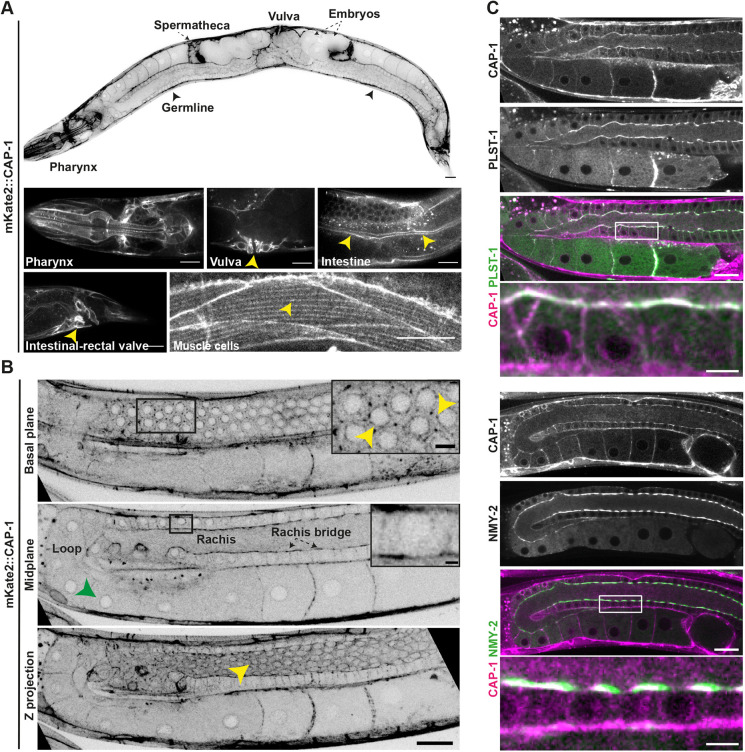
**CAP-1 localizes at actomyosin-enriched structures in the *C. elegans* germline.** (A) Top: Inverted greyscale confocal fluorescence image of an adult *C. elegans* hermaphrodite expressing CAP-1 endogenously tagged with mKate2. Arrows point to spermatheca and embryos. Arrowheads indicate germline. Scale bar: 20 µm. Bottom: Representative confocal fluorescence images of mKate2::CAP-1 localization in the pharynx, vulva, intestine, rectal valve and muscle striations. Arrowheads indicate CAP-1 localization in respective tissues. Scale bars: 20 µm. (B) Representative inverted greyscale confocal fluorescence images of the *C. elegans* germline expressing mKate2::CAP-1. Basal plane view showing localization at the germ cell cortex (yellow arrowheads), midplane view showing enrichment of CAP-1 at rachis bridges and nuclear envelope of germ cells (green arrowhead), and a maximum intensity *z*-projection view showing CAP-1-enriched actomyosin corset in the gonad (yellow arrowhead). Insets show enlarged views of the boxed areas. Scale bars: 20 µm (main panels); 5 µm (insets). (C) Midplane views of the *C. elegans* germline showing localization of mKate2::CAP-1 with PLST-1::GFP and NMY-2::GFP. Scale bars: 20 µm. White boxes mark the magnified view of the rachis region below. Scale bars: 5 µm.

To explore the expression pattern and subcellular localization of CAP-1 in the adult *C. elegans* hermaphrodite, we generated a strain in which CAP-1 is endogenously tagged with the fluorescent protein mKate2 at its N terminus. The brood size (Fig. S1A) and embryonic viability (N2, 99.9% viable embryos, *n*=813 embryos analysed; mKate2::CAP-1, 99.4% viable embryos, *n*=716 embryos analysed) of this strain were not different from wild-type worms, indicating that CAP-1 function was not perturbed by its tagging. Imaging by spinning-disc confocal microscopy revealed that CAP-1 is expressed in multiple adult tissues, including the germline, spermatheca, pharynx and vulva. It was also found to localize at the apical side of gut epithelial cells, in striations along body-wall muscle cells and in the intestinal-rectal valve (Fig. 1A).

The *C. elegans* gonad, made of two symmetrical U-shaped arms, contains a syncytium of germ cells, which first form sperm that is stored in the spermatheca and later form oocytes (Kimble and Crittenden, 2007). Germ cells are arranged in a hexagonal pattern around a common cytoplasm-filled core called the rachis, to which they are connected via rings known as rachis bridges (Amini et al., 2014; Bauer et al., 2021; Zhou et al., 2013). We found CAP-1 to be expressed in all germ cells, where it localized to cell cortices and was highly enriched at rachis bridges and within the actomyosin corset surrounding the rachis (Fig. 1B, yellow arrowheads). CAP-1 was also observed surrounding the germ cell nuclei (Fig. 1B, green arrowhead). The perinuclear localization is most likely explained by CP being a component of the dynactin complex (Hammesfahr and Kollmar, 2012; Hodgkinson et al., 2005), because it is known that dynein localizes at the nuclear envelope (Malone et al., 2003; Zhou et al., 2009). We further investigated the spatial relationship between CAP-1 and other components of the actomyosin corset (Priti et al., 2018). We crossed the mKate2::CAP-1 strain with strains expressing endogenously GFP-tagged PLST-1/plastin, an actin-crosslinker and NMY-2/non-muscle myosin II. As shown in Fig. 1C, CAP-1 colocalized with PLST-1 and with NMY-2 along the rachis, confirming that it is an integral component of the actomyosin corset in the syncytial germline.

### Precise CAP-1 levels are required for maintenance of germline architecture

Given the prominent localization of CAP-1 in the germline, we focused on understanding the role of CAP-1 in that tissue. We generated a *cap-1* null mutant using CRISPR-Cas9 to delete the entire *cap-1* gene. However, the homozygous null mutants obtained from heterozygous mothers, although successfully accomplishing embryogenesis, arrested as larva. Because the homozygous *cap-1* mutants did not reach adulthood, we sought to understand its role through RNA interference (RNAi) starting from the first larval stage (L1). The degree of CAP-1 knockdown (KD) was assessed by performing *cap-1* RNAi in worms expressing mKate2::CAP-1 and a membrane marker (GFP::PLC1δ-PH) (Fig. S1B). Normalized relative to the membrane marker, we measured an average ∼60% reduction in CAP-1 fluorescence in the *cap-1(RNAi)* germlines compared with control (Fig. S1C).

**Fig. 2.**
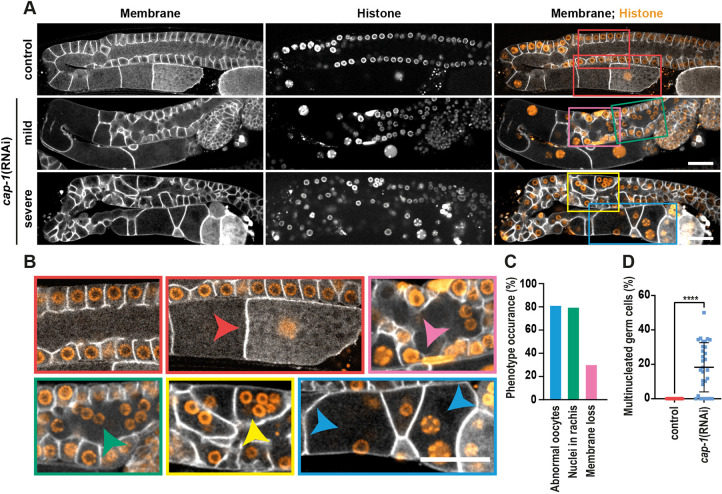
**CAP-1 is required for the maintenance of germline architecture and functionality.** (A) Confocal fluorescence images of the *C. elegans* germline expressing a membrane marker, GFP::PLC1δ-PH, and a nuclear marker, HIS-58::mCherry. Top row shows control germlines, and middle and bottom rows show *cap-1(RNAi)* germlines with mild and severe phenotypes. (B) Enlarged views of the colour-coded boxed areas from A. Control germline with streamlined peripheral germ cell nuclei and normal oocyte morphology (red boxes). *cap-1(RNAi)* germline showing regions with membrane loss (pink box), nuclei mispositioned within the rachis (green box and green arrowhead), multinucleated germ cells (yellow box and yellow arrowhead), and abnormal oocyte with multiple nuclei (blue box and blue arrowheads). Scale bar: 20 µm. (C) Percentage of gonads showing the occurrence of the phenotypes ‘abnormal oocytes’, ‘nuclei in rachis’ and ‘membrane loss’ in *cap-1(RNAi)* worms (with either mild or severe phenotype). The colour of the columns matches the colour of the arrows in B. (D) Percentage of multinucleated germ cells in gonads of control (*n*=32) versus *cap-1(RNAi)* (*n*=30) worms. ****P<0.0001 (unpaired Student's *t*-test).

Depletion of CAP-1 in worms co-expressing a membrane marker and a nuclear marker (HIS-58::mCherry) resulted in a range of phenotypes with varying degrees of severity and penetrance, covering all regions of the gonad. Fig. 2A provides examples of ‘mild’ and ‘severely’ affected gonads displaying the following phenotypes: germ cell membrane loss, multinucleated germ cells and abnormally sized oocytes, as reported in a previous study (Green et al., 2011), and nuclei suspended in the rachis. Enlargements in Fig. 2B highlight the defects. Quantification of the frequency of appearance of these phenotypes in the entire *cap-1(RNAi)* population is shown in Fig. 2C,D. Consistently, approximately 50% of the worms (*n*=57) had severe germline defects, were sterile and did not produce any embryos.

**Fig. 3.**
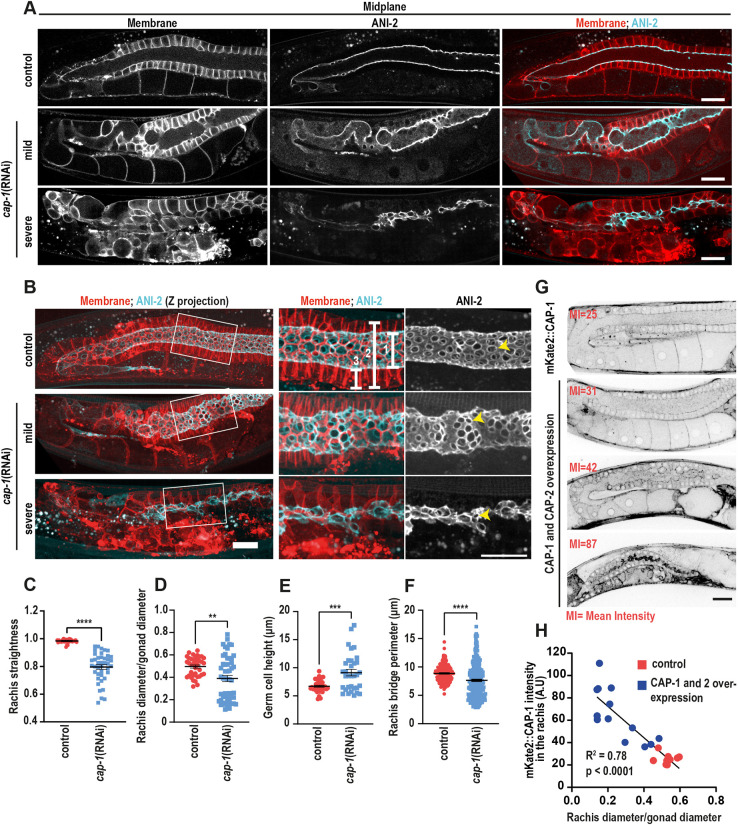
**Depletion as well as overexpression of CAP-1 leads to defects in germline architecture.** (A) Midplane confocal fluorescence images of gonads expressing a membrane marker, mCherry::PLC1δ-PH, and a rachis bridge marker, ANI-2::GFP, for control (top), mildly (middle) and severely (bottom) affected *cap-1(RNAi)* worms. Scale bar: 20 µm. (B) *z*-projected views of the gonads shown in A. Right: Magnified views of the regions marked by white boxes in the left panel. Scale bars: 5 µm. (C-F) Quantification of rachis straightness, rachis width normalized to gonad width, calculated as the ratio of the length of line 1 to line 2 (white brackets in B), germ cell height (length of bracket 3 in B) and rachis bridge perimeter (yellow arrowheads in B) in the gonads of control (*n*=38) versus *cap-1(RNAi)* (*n*=41) worms. **P<0.01, ***P<0.001, ****P<0.0001 (unpaired Student's *t*-test). (G) Inverted greyscale confocal fluorescence images of gonads of mKate2::CAP-1- and CAP-2-overexpressing worms. Mean intensity (MI) of mKate2::CAP-1 fluorescence measured at the rachis is annotated in red. Scale bar: 20 µm. Quantification of fluorescence intensity at the rachis bridge was measured by drawing a 5-pixel-wide line across the rachis bridges. (H) Correlation between mKate2::CAP-1 intensity versus normalized rachis width in control worms, and worms overexpressing CAP-1 and CAP-2. Simple linear regression analysis was carried out to calculate the correlation coefficient (R^2^). A.U., arbitrary unit.

Because CAP-1 localizes prominently to the actomyosin corset surrounding the rachis, we sought to determine the effect of CAP-1 KD on rachis morphology. We performed *cap-1* RNAi on a strain expressing the anillin isoform ANI-2 tagged with GFP, to mark the rachis bridges (Amini et al., 2014), and a membrane marker (mCherry::PLC1δ-PH). In contrast with the rachis of control gonads, which was straight and maintained a fairly constant diameter, the rachis of *cap-1(RNAi)* worms was meandering and of variable diameter (Fig. 3A,B). We quantified rachis straightness by dividing the length of a straight line between the two ends of the rachis by the length of a path along the centre of the rachis. In control worms, the rachis straightness was close to one, but it was significantly reduced upon *cap-1* KD (Fig. 3C). Next, we measured the ratio between rachis and gonad diameters. In *cap-1(RNAi)* worms, the variance was higher than control, i.e. there were both gonads with wider than normal and gonads with narrower than normal rachides (Fig. 3D). However, 50% of *cap-1(RNAi)* gonads (*n*=57) exhibited a severely constricted rachis compared with the control (Fig. 3D,E, Movie 1). The decrease in rachis width was accompanied by an increase in germ cell height (Fig. S1D). Also, the perimeter of rachis bridges was significantly reduced after the depletion of CAP-1 (Fig. 3F). Interestingly, *cap-1^+/−^* deletion heterozygous mutants did not show any defect in the germline compared with control (Fig. S1E-G), suggesting that the maternal CAP-1 level is sufficient for proper germline development.

To determine whether the germline defects observed were due exclusively to *cap-1* depletion in the germline or whether *cap-1* depletion in other tissues might contribute to the phenotype, we performed *cap-1* RNAi in a germline-specific RNAi strain (DCL569), in which a null mutation in the RNAi-essential gene *rde-1* is rescued only in the germline by expression of *rde-1* under a germline-specific promoter (Zou et al., 2019). Following *cap-*1 RNAi in this strain, we observed the same germline defects as we found previously with whole worm RNAi (Fig. S1H), with approximately 75% penetrance, suggesting a tissue-autonomous role of CAP-1 in maintaining germline structure.

Finally, we tested the effect of CP overexpression in the germline by injecting mKate::*cap-1* and *cap-2* on a bicistronic expression cassette, under the *pie-1* germline-specific promoter, into the strain with endogenous *mKate2::cap-1*. By measuring the CAP-1 fluorescence intensity, we identified a few F1 progeny of the injected worms with up to threefold CAP-1 overexpression compared with control, as well as worms in which silencing of *cap-1* occurred. Importantly, in worms overexpressing CP the gonad was much smaller than normal, the rachis was severely constricted, oocyte formation was defective, and the worms were sterile (Fig. 3G). Rachis width in these worms was negatively correlated with mKate2::CAP-1 intensity at the rachis, even when normalized to gonad diameter (Fig. 3H).

In summary, both partial depletion and overexpression of CP specifically within the germline led to major structural defects, with adverse effects on fecundity. Thus, CP function appears to be required for normal germline architecture in the adult hermaphrodite.

### CAP-1 is necessary for germline structural maintenance, but not for germline formation

**Fig. 4.**
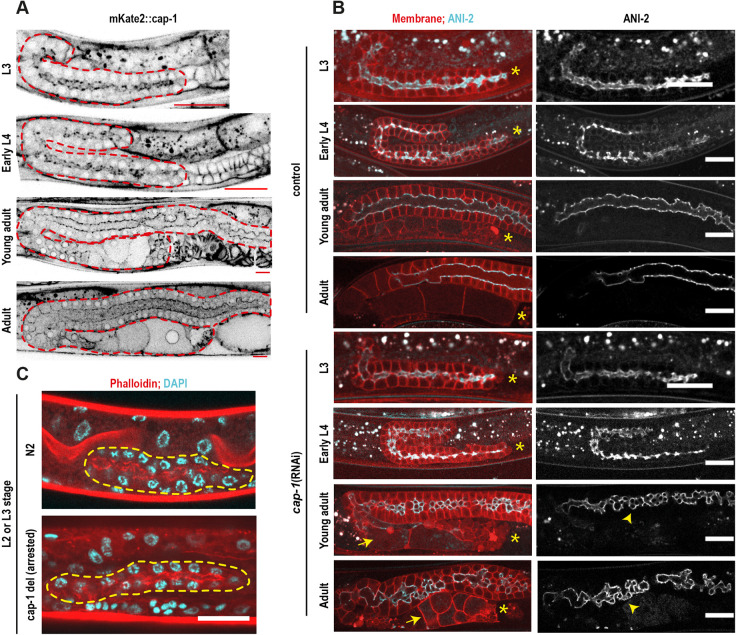
**CAP-1 expression and knockdown during germline morphogenesis.** (A) Inverted greyscale confocal fluorescence images of germlines expressing mKate2::CAP-1 at late larval stages and adulthood. Red dashed lines mark the boundaries of the gonads. Scale bars: 20 µm. (B) Confocal fluorescence images of germlines expressing the membrane marker (red) and ANI-2::GFP (cyan) from larval stage 3 until adulthood in control and *cap-1(RNAi)* worms. Asterisks indicate the proximal end of the germlines. Arrows and arrowheads indicate defective oocytes and rachis, respectively in *cap-1(RNAi).* Scale bars: 20 µm. (C) Confocal fluorescence images of L2 or L3 stage control and *cap-1* null mutant germlines stained with phalloidin, an F-actin marker and DAPI, a chromatin marker. Yellow dashed lines outline the gonad. Scale bar: 20 µm.

To establish whether CAP-1 function is required during germline morphogenesis and/or for maintenance of its structure, we followed germline formation in *mKate2::cap-1* worms and cap*-1(RNAi)* conditions from the L3 stage until adulthood (Fig. 4). CAP-1 fluorescence was visible in the rachis from the early larval stages (Fig. 4A), yet we did not observe any defect in gonad morphology during larval development in *cap-1* KD worms (Fig. 4B). Starting from the young adult stage we observed the onset of mild defects in gonad morphology in *cap-1*-depleted worms, mostly oocyte morphology defects, and the defects increased in severity with time resulting in gross structural defects in the adult germline. To rule out the possibility of inefficient KD in the early larval stages, we analysed the gonads in *cap-1* null mutants arrested at the L2 or L3 stage. Consistent with the RNAi experiments, gonad formation was unaffected in the arrested larvae (Fig. 4C). These results suggest that CAP-1 is not required during gonad development, but is essential for maintenance of its structure during adulthood.

### CAP-1 depletion leads to a CYK-1-dependent increase in F-actin in the germline

**Fig. 5.**
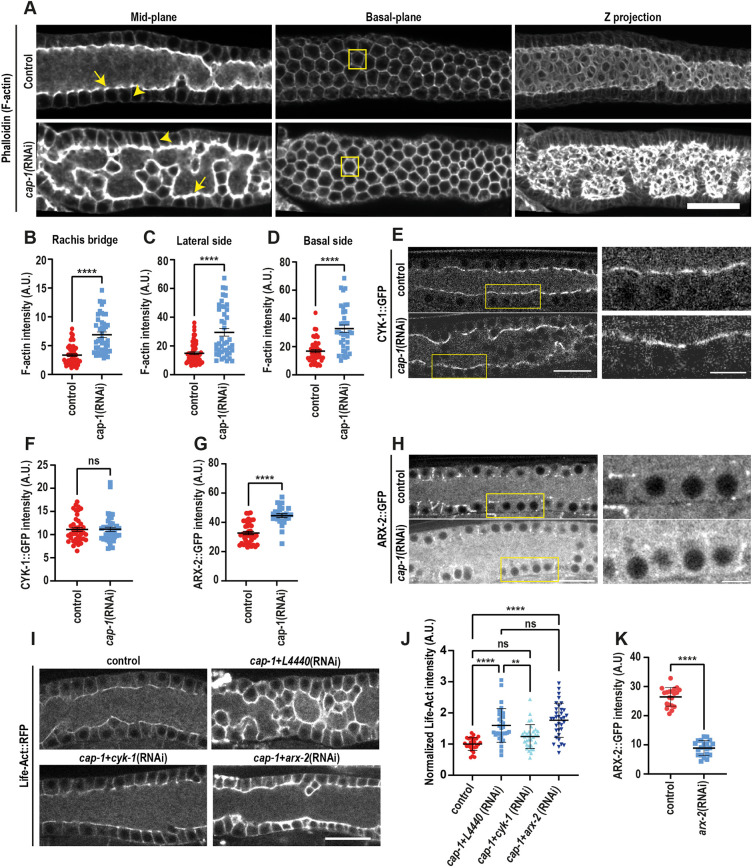
**CAP-1 regulates F-actin levels in the *C. elegans* germline through CYK-1.** (A) Midplane, basal plane and maximum-intensity *z*-projected images of phalloidin-stained germlines in control and *cap-1(RNAi)* worms. Scale bar: 20 µm. (B-D) Quantification of mean F-actin intensity in untreated control (*n*=52) versus *cap-1(RNAi)* (*n*=42) worms at rachis bridges (arrows in A), lateral membranes (arrowheads in A) and basal sides of the germ cells (boxes in A). Quantification of fluorescence intensity at the rachis bridge was measured by drawing a 5-pixel-wide line across the rachis bridges. Hexagonal germ cell boundaries were traced using a 5-pixel-wide line at the basal plane and the cell junctions at the lateral plane to measure the intensity at lateral sides and basal sides of the germ cells, respectively. (E) Confocal fluorescence images of germlines expressing CYK-1::GFP in control versus *cap-1(RNAi)* worms. Right-hand panels show magnified views of the rachis region (marked by yellow boxes). Scale bars: 20 µm (left); 5 µm (right). (F) Quantification of CYK-1::GFP intensity at the rachis bridges in control (*n*=41) versus *cap-1(RNAi)* (*n*=40) worms. (G) Quantification of ARX-2::GFP intensity at the rachis bridges in control (*n*=41) versus *cap-1(RNAi)* (*n*=40) worms. Total ARX-2::GFP fluorescence was calculated by tracing the germline outline at the midplane in the meiotic region. (H) Confocal fluorescence images of germlines expressing ARX-2::GFP in control versus *cap-1(RNAi)* worms. Right-hand panels show magnified views of the rachis region (marked by yellow boxes). Scale bars: 20 µm (left); 5 µm (right). (I) Confocal fluorescence images of germlines expressing LifeAct::RFP in control, *cap-1(RNAi)*, *cap-1(RNAi);cyk-1(RNAi)* and *cap-1(RNAi);arx-2 (RNAi)* worms. Scale bar: 20 µm. (J) Quantification of Life-Act intensity, normalized to the fluorescence intensity of a membrane marker, following KD of CAP-1, CAP-1 with CYK-1, and CAP-1 with ARX-2. (K) Quantification of ARX-2::GFP intensity following ARX-2 depletion by RNAi. Total ARX-2::GFP fluorescence was calculated by tracing the germline outline at the midplane in the meiotic region. **P<0.01; ****P<0.0001; ns, not significant (unpaired Student's *t*-test in B-D,F,G,J,K; one-way ANOVA test in J). A.U., arbitrary unit.

One of the main functions of CP is to regulate the growth of actin filaments (Edwards et al., 2014). To examine the relationship of CAP-1 with F-actin in *C. elegans*, we co-stained mKate2::CAP-1 worms with the F-actin marker phalloidin. We found that CAP-1 colocalized with most, but not all, F-actin structures (Fig. S2). CAP-1 was absent from spermathecal stress fibres, but colocalized with F-actin in the pharynx, intestine and germline. Next, we tested the effect of *cap-1* RNAi on the germline actin cytoskeleton with phalloidin staining of dissected gonads (Fig. 5A). For this purpose, we examined worms with a relatively mild phenotype, because germline structure in the severely affected worms was too disrupted to compare with controls. In control gonads, F-actin was observed at the basal (marked by yellow box, Fig. 5A) and lateral cortices of the germ cells (yellow arrowheads, Fig. 5A) and was particularly enriched at rachis bridges (yellow arrows, Fig. 5A). Following *cap-1* RNAi, phalloidin staining increased significantly (Fig. 5A). Quantification of fluorescence intensities in specific subcellular regions showed that F-actin increased by approximately twofold at the rachis bridges, and lateral and basal sides of the germ cells (Fig. 5B-D). A similar increase was also observed with the endogenously GFP-tagged F-actin-bundling protein PLST-1 in live worms (Fig. S3A-D).

Given the substantial increase in F-actin levels in the germline, we explored the localization and expression levels of two well-known germline actin nucleators, the formin CYK-1 and the Arp2/3 complex, in control and *cap-1* KD conditions, using endogenous fluorescent markers. As reported before, CYK-1::GFP was primarily found at the rachis (Priti et al., 2018), and we did not observe any difference in the localization or levels of CYK-1 in the germline following *cap-1* RNAi (Fig. 5E,F). The Arp2/3 component ARX-2::GFP appeared as sparse puncta along the rachis and throughout the germ cell cortices, and following *cap-1* RNAi its level increased by 30% throughout the germline (Fig. 5G,H).

Next, we sought to determine the contribution of each of these polymerization-promoting factors to the increase in F-actin following *cap-1* KD. To this end, we performed double RNAi experiments, depleting *cap-1* along with either *cyk-1* or *arx-2* and monitored the change in F-actin with LifeAct::RFP. The level of F-actin in the *cap-1; cyk-1* double KD was not significantly different from the empty vector control, suggesting that CYK-1 activity is required for the increase in F-actin observed in *cap-1* KD diluted with empty vector (Fig. 5I,J). In contrast, the level of F-actin in the *cap-1; arx-2* double KD was significantly increased compared with the control, similar to the *cap-1* single (diluted) KD, suggesting that the Arp2/3 complex is not responsible for the increase in F-actin in *cap-1(RNAi)* germlines (Fig. 5I,J). We confirmed that *arx-2(RNAi)* reduced endogenous *arx-2* by 66% by quantifying the fluorescence of endogenous ARX-2::GFP in control and *arx-2(RNAi)* germlines (Fig. 5K). Taken together, these results show that the increase in polymerized actin within the germline following partial depletion of CP is dependent on the formin CYK-1.

### CAP-1 depletion leads to an increase in active NMY-2 and tension at the germline corset

**Fig. 6.**
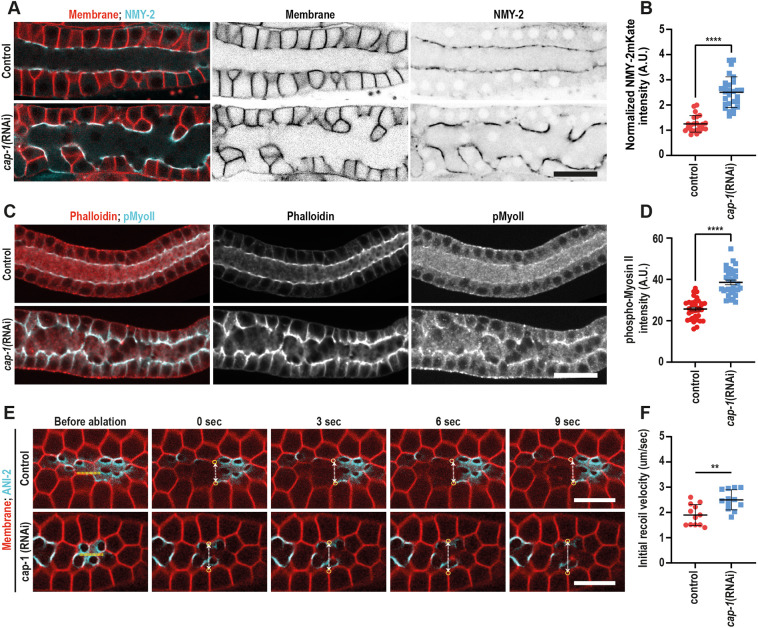
**CAP-1 depletion leads to an increase in myosin activity and tension at the rachis.** (A) Confocal images of the germline expressing NMY-2::GFP (cyan) and GFP::PLC1δ-PH (red) in control and *cap-1(RNAi)* worms. Non-merged images are in inverted greyscale. Scale bar: 20 µm. (B) Quantification of NMY-2::GFP intensity at the rachis bridge in control (*n*=23) versus *cap-1*(RNAi) (*n*=24) worms. ****P<0.0001 (unpaired Student's *t*-test). (C) Confocal images of the germline in control and *cap-1*(*RNAi)* worms stained for phosphorylated myosin II (pMyoII, cyan) and F-actin (phalloidin, red). Scale bar: 20 µm. (D) Quantification of phospho-myosin II intensity at the rachis bridge in control (*n*=35) versus *cap-1(RNAi)* (*n*=31) worms. ****P<0.0001 (unpaired Student's *t*-test). (E) Time-lapse images of laser incision in control versus *cap-1(RNAi)* worms expressing a membrane marker, mCherry::PLC1δ-PH, and a rachis bridge marker, ANI-2::GFP. Laser cut is indicated by a dashed yellow line. White dashed arrows show displacement between the two vertices marked by yellow circles. Scale bars: 5 µm. (F) Quantification of initial recoil velocity in the rachis of control (*n*=12) versus *cap-1(RNAi)* (*n*=11) worms. Quantification of fluorescence intensity at the rachis bridge (B,D) was measured by drawing a 5-pixel-wide line across the rachis bridges. **P<0.01 (unpaired Student's *t*-test). A.U., arbitrary unit.

Given the increase in F-actin at the rachis following *cap-1* depletion and the known dependence of syncytial germline architecture on rachis actomyosin contractility, we investigated the effect of *cap-1* RNAi on non-muscle myosin II/NMY-2 at the rachis. As shown in Fig. 6A, NMY-2::GFP was concentrated along the rachis bridges in both control and *cap-1(RNAi)* worms and there was a twofold increase in NMY-2 levels at the rachis following partial CAP-1 depletion (Fig. 6A,B). Furthermore, using an antibody that recognizes only the phosphorylated, i.e. the active form, of Myosin II, we also observed an increase in the levels of phospho-myosin II at the rachis (Fig. 6C,D), suggesting an increase in myosin activity within the actomyosin corset in *cap-1(RNAi)* conditions.

It is not possible to measure tension directly within the actomyosin corset. However, a widely accepted method for assessing tension in the cortex is to measure the speed of retraction following laser incision (Saha et al., 2016). We therefore performed laser cuts at the rachis surface and followed the recoil dynamics of the remaining cell edges (Fig. 6E, Movie 2). Consistent with the increase in active myosin, quantification of initial recoil velocity showed a statistically significant increase in recoil velocity in the *cap-1(RNAi)* condition compared with control (Fig. 6F).

The observed increase in NMY-2 could, in theory, be due to more F-actin-binding sites, slower NMY-2 turnover, or a combination of both. In order to examine whether the dynamics of NMY-2 were affected by *cap-1* depletion, we carried out fluorescence recovery after photobleaching (FRAP) on endogenously tagged NMY-2::GFP at the rachis bridge. We did not observe a significant difference in the recovery kinetics of NMY-2 in *cap-1(RNAi)* conditions versus the control, although we did observe a trend towards a larger immobile fraction in *cap-1*-depleted gonads (Fig. S4A-D). Overall, we found an increase in active NMY-2 and an increase in actomyosin corset contractility following CP depletion.

### Simulations of actomyosin networks, with random architectures, suggest increased myosin levels as the main driver of enhanced contractility as a result of CAP-1 partial loss of function

**Fig. 7.**
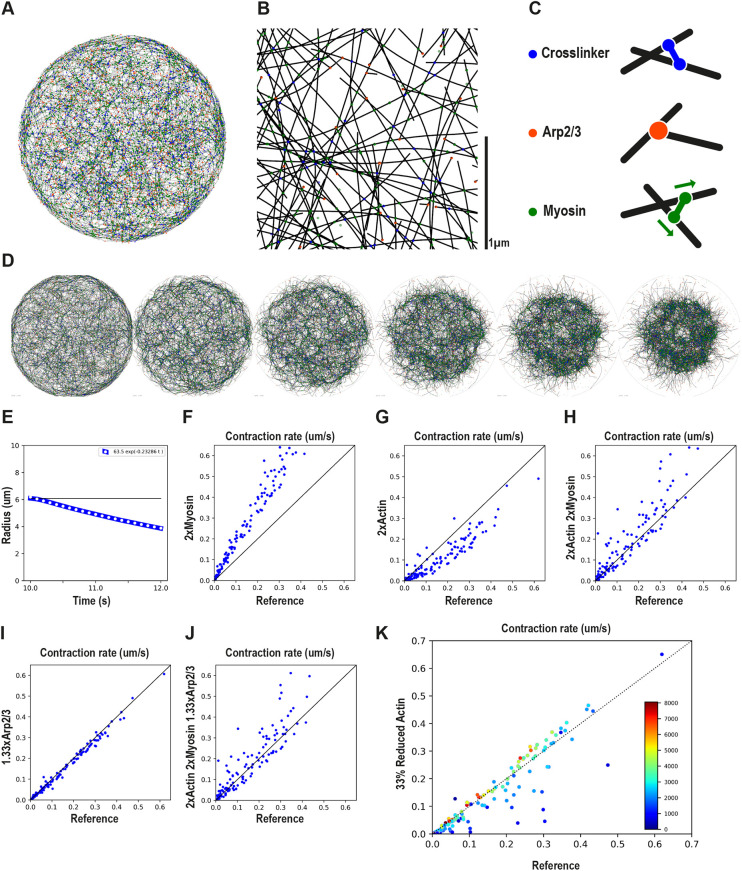
**Simulation of actomyosin network with random architectures.** (A) A circular network of radius 6 µm was simulated in 2D with actin filaments (black), crosslinkers (blue), Arp2/3 (brown) and myosin motors (green). (B) An enlarged portion of the network shown in A. (C) Filaments are connected in three possible ways. Crosslinkers are passive Hookean links with attachment points that are fixed on the filaments. Arp2/3 are also fixed on the filaments but induce both a translational and rotational link that constrain the daughter filament to branch off the mother filament with an angle of 72°. The myosin motor is represented with two motor units that can move along the filaments towards the plus/barbed ends. Assumptions of the models are described in the supplementary Materials and Methods. (D) The network shown in A is contractile owing to the work of myosin motors in the presence of crosslinkers. Snapshots separated by 0.4 s are shown. (E) The radius covered by the network is extracted automatically from the coordinates of the filaments at each time point (see supplementary Materials and Methods). The contraction rate is then derived by fitting an exponential. White dots indicate the exponential fit over the calculated radius (blue line). The horizontal black line indicates the initial size of the network. (F-J) In order to evaluate the effect on contraction rate of doubling the amount of myosin motors (F) or F-actin (G) or both (H) or increasing Arp2/3 by 33% (I) or all of them together (J), 128 random networks were considered. Each blue dot represents the outcome of two simulations: the reference contraction rate is used for the *x* coordinates, and the contraction rate derived with the change in myosin and/or F-actin and/or Arp2/3 is used for the *y* coordinates. (K) For simulating CP overexpression in Cytosim, the polymer mass was decreased by 30% in all random networks. The dots are colour-coded for polymer density (blue=sparse to red=dense). Dots above the reference line represent simulations with increased contractility, whereas dots below the line have reduced contractility.

The KD of CAP-1 changed several parameters of the actomyosin network: both F-actin and myosin were roughly doubled, and Arp2/3 was also increased by ∼30%. At the same time, constricted rachis morphology and laser incision experiments pointed to an increase in cortical tension. However, determining the cause and effect in this situation is complicated, because all the parameters changed by CAP-1 depletion could, in principle, influence the contractility of the system. The quantity of myosin motors recruited to the cortex would naturally affect its contractile properties, but the mesh size determined by the actin density is also expected to influence contractility. Furthermore, *in vitro* reconstitution assays and modelling have shown that the balance between linear and branched actin networks influences the level of ‘network connectivity’ and therefore its contractility (Ennomani et al., 2016). To investigate the problem further, we turned to stochastic simulations of 2D patches of cortex using Cytosim (Nedelec and Foethke, 2007) (Fig. 7A,B). The patch was connected by crosslinkers and Arp2/3 and contained both linear and branched polymers (Fig. 7C). Filaments have bending elasticity, and connectors, represented by Hookean springs, bind and unbind stochastically to the filaments. The movements of motors connecting multiple filaments drive contractility, which occurs only in the presence of passive crosslinkers and because filaments may bend (Belmonte et al., 2017). Although their geometry is different, the retraction of a patch of cortex in the simulation is analogous to the retraction of the cortex following an experimental laser incision. One can imagine that the onset of the simulation is immediately after a circular laser cut extracted the cortical patch from the larger cortex.

The model, its assumptions and procedure are described in the Materials and Methods section. Because we do not know the exact characteristics of the actomyosin network in the *C. elegans* germline rachis, we have generated 128 arbitrary network architectures, by randomly varying some key parameters of the system, such as the number of nucleators, Arp2/3, motors and crosslinkers (see Materials and Methods and Fig. S5A for examples). For each network architecture, we derived the contraction rate after the addition of motors (Fig. 7D,E, Movie 3). We then systematically applied the perturbations derived from CAP-1 KD: (1) doubling of F-actin, (2) doubling of myosin and (3) 30% more Arp2/3, as well as combinations of the above. We then compared the contraction rate of each architecture with the same architecture subjected to one or more of the perturbations.

A clear outcome of these simulations was that doubling the amount of myosin motors always increased contractility (Fig. 7F). Second, we found that doubling the amount of F-actin, on its own, most often decreased contractility, but on some occasions increased it (Fig. 7G). Applying the two modifications together led to mixed effects, but most often led to increased contractility (Fig. 7H). Finally, a 33% increase in Arp2/3 did not significantly change contractility (Fig. 7I,J). The results were remarkably consistent for all network architectures, although these architectures ranged from sparse to dense and were visually different (Fig. S5A,B). The simulations clearly indicated that the recruitment of myosin enhances contractility, and that this effect alone could explain the increase in contractility observed in CAP-1 KD.

Finally, to simulate the effect of CP overexpression, we shortened the filaments by reducing the amount of polymer available to form the network by one-third. The simulations showed that if the network density is high, this reduction increases contraction (Fig. 7K). In contrast, if the network density is low, shortening the filaments will prevent them from crossing and to form a percolated network, and in that case the system is no longer able to contract uniformly. We expect natural networks to be dense, and these results are thus consistent with our experimental results showing a highly constricted rachis when CP was overexpressed (Fig. 3G,H).

### Inhibiting actomyosin contractility can prevent rachis constriction in the CP KD condition

**Fig. 8.**
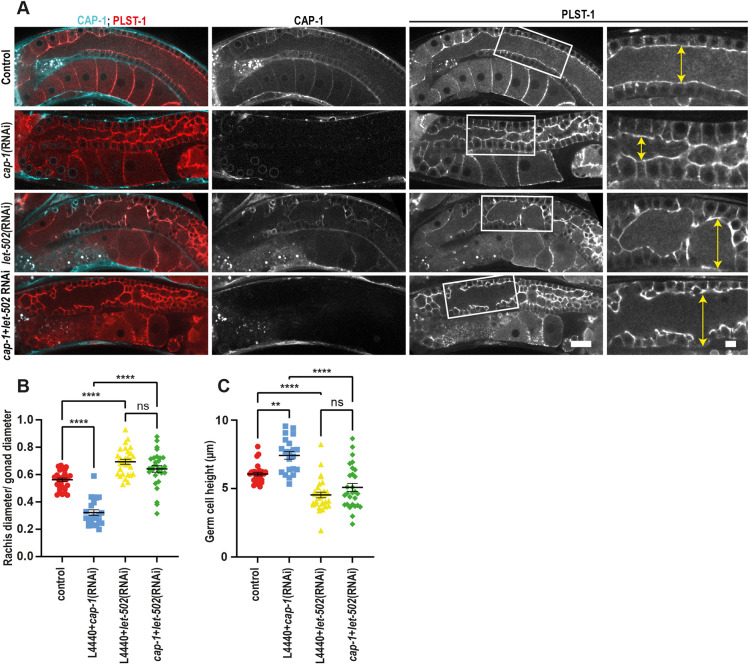
**LET-502 depletion mitigates constricted rachis phenotype in gonads of *cap-1(RNAi)* worms.** (A) Midplane confocal views of the whole germline and magnified views of the rachis region (white boxes) in worms expressing PLST-1::GFP (red) and mKate2::CAP-1 (cyan) in control, *cap-1(RNAi)*, *let-502(RNAi)* and *cap-1(RNAi);let-502(RNAi)* worms*.* Double-headed arrows mark rachis diameter. Scale bars: 20 µm (main panels); 5 µm (magnified images). (B,C) Quantification of rachis width and germ cell height in germlines of control (*n*=28), *cap-1(RNAi)* (*n*=23), *let-502(RNAi)* (*n*=31) and *cap-1(RNAi);let-502(RNAi)* (*n*=29) worms. **P<0.01; ****P<0.0001 (one-way ANOVA test). ns, not significant.

**Fig. 9.**
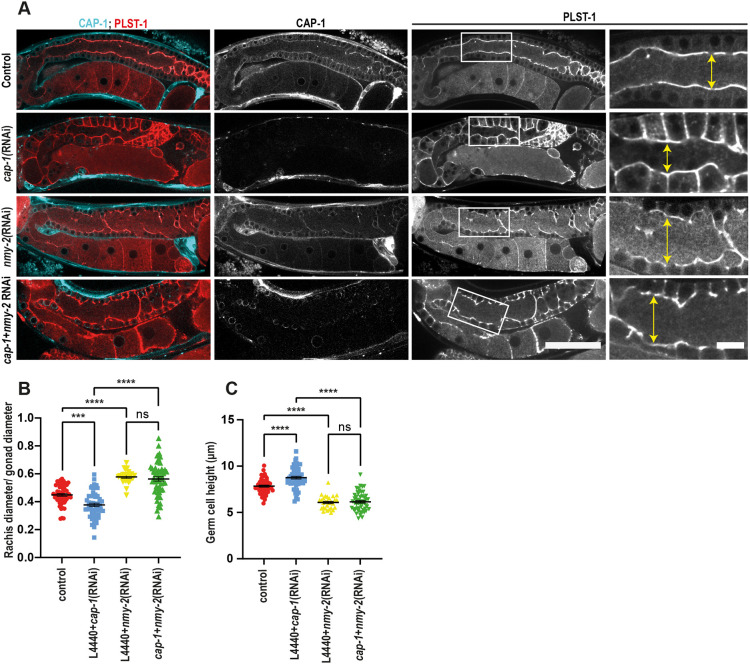
**Partial depletion of NMY-2 leads to a wider rachis in gonads of *cap-1(RNAi)* worms.** (A) Midplane confocal views of the whole germline and magnified views of the rachis region (white boxes) in worms expressing PLST-1::GFP (red) and mKate2::CAP-1 (cyan) in control, *cap-1(RNAi)*, *nmy-2(RNAi)* and *cap-1(RNAi);nmy-2(RNAi)* worms*.* Double-headed arrows mark rachis diameter. Scale bars: 50 µm (main panels); 10 µm (magnified images). (B,C) Quantification of normalized rachis width and germ cell height in germlines of control (*n*=53), *cap-1(RNAi)* (*n*=55), *nmy-2(RNAi)* (*n*=25) and *cap-1(RNAi);nmy-2(RNAi)* (*n*=57) worms. ***P<0.001; ****P<0.0001 (one-way ANOVA test). ns, not significant.

Both experimental evidence and simulation results indicated that CAP-1 depletion leads to an increase in rachis contractility. We hypothesized that the increase in contractility was responsible for at least some of the phenotypes present in *cap-1(RNAi)* gonads*.* To test this idea, we first compared germline morphology of *mel-11(RNAi)* with *cap-1(RNAi)* worms. MEL-11 is the *C. elegans* myosin phosphatase, and its depletion is known to lead to an increase in actomyosin contractility (Piekny and Mains, 2002). Notably, we observed similar morphological changes, namely a constricted rachis and increased germ cell height in *cap-1(RNAi)* and *mel-11(RNAi)* (Fig. S6). To test whether *cap-1* germline defects depend on increased rachis contractility, we depleted the myosin activator Rho kinase/LET-502 in *cap-1(RNAi)* worms. Endogenously tagged LET-502 is expressed throughout the germline with partial enrichment at the rachis (Fig. S7A). As evident in Fig. 8, *let-502(RNAi)* worms displayed a phenotype opposite of that of *mel-11(RNAi)* or *cap-1(RNAi)*, namely it had a wider than normal rachis and shorter germ cell membranes. We performed the double RNAi KD in worms expressing mKate2::CAP-1 and quantified CAP-1 intensity to confirm *cap-1* depletion and rule out a dilution effect (Fig. S7B). Germlines with double KD of *let-502* and *cap-1* resembled the *let-502(RNAi)* phenotype with a broad rachis and short germ cell membranes (Fig. 8). To confirm that the defects observed in *cap-1(RNAi)* gonads are due to increased contractility and not because of a different downstream target of LET-502, we carried out partial depletion of NMY-2 in *cap-1(RNAi)* gonads. Similar to the LET-502 results, gonads depleted of both NMY-2 and CAP-1 had wider rachides and shorter germ cells, phenocopying partial depletion of NMY-2 (Fig. 9). We confirmed that *cap-1(RNAi*) and *nmy-2(RNAi*) gonads showed significant depletion of endogenously tagged CAP-1 and NMY-2 expression, respectively, in parallel KD experiments (Fig. S7C-E). Together, these results indicate that the rachis architecture defects observed in *cap-1(RNAi)* gonads require increased contractility, mediated by LET-502-dependent phosphorylation of NMY-2 in the actomyosin corset of the syncytial germline.

## DISCUSSION

The expression pattern of endogenously tagged CAP-1 shows that CP functions in multiple adult tissues, including muscle and pharynx, as well as in embryos, and makes *C. elegans* an excellent model in which to study the developmental and physiological function of CP. Here, we focused on the role of CP in the germline, where we found it to be particularly enriched in the actomyosin corset surrounding the rachis of the syncytial germline. Both KD and overexpression of CP resulted in severe perturbation to gonad architecture, leading us to conclude that precise levels of CP are needed to maintain the intricate structure of the syncytial gonad. Complex effects of depleting or adding capping protein were expected based on *in vitro* studies, which showed that, although CP caps elongating actin filaments, it also increases the frequency of Arp2/3-mediated nucleation events in a dendritic network and therefore the amount of CP determines the architecture of the actin network (Akin and Mullins, 2008).

Some of the germline defects that we observed in *cap-1(RNAi)* worms have been previously reported in a high-throughput screen (Green et al., 2011); however, the function of CP in germline architecture was not investigated. We found that depletion of CAP-1 led to a twofold increase in F-actin and myosin II levels at the rachis surface. Given the canonical role of CP to cap actin filaments and stop their polymerization, it is not surprising that when its levels are reduced by 60% there is a twofold increase in filamentous actin. Interestingly, we found this increase in F-actin to be entirely dependent on the formin CYK-1 and independent of the Arp2/3 complex, despite the fact that Arp2/3 levels increased upon *cap-1* RNAi, whereas CYK-1 levels remained constant. These findings suggest that the actin cytoskeleton at the rachis is primarily polymerized by CYK-1, a conclusion that is consistent with the prominent localization of CYK-1 at rachis bridges. Arp2/3, in contrast, is not enriched at the rachis, and so the increase in cytoplasmic Arp2/3 following *cap-1* depletion might have an effect on cytoplasmic actin networks in the germline.

It is well established that formin and CP compete for binding at the barbed end (Shekhar et al., 2015). Therefore, in the absence of its competitor, CYK-1 can polymerize longer filaments. The bias toward formin-mediated actin polymerization in the absence of CP suggests that CP in the germline acts to regulate the balance between linear and Arp2/3-mediated branched networks and thus affects the architecture of the actin cytoskeleton. This is reminiscent of fission yeast, in which CP associates with actin patches to exclude formin from the Arp2/3-mediated actin structures, hence preserving the identity of distinct actin structures (Billault-Chaumartin and Martin, 2019). The architecture of the network determines its connectivity and mechanical properties. Network connectivity affects myosin-mediated contractility according to a bell-shaped curve (Belmonte et al., 2017; Ennomani et al., 2016). We do not know where along this connectivity curve the rachis actomyosin network is in wild-type conditions, but it is possible that a decrease in connectivity due to the decreased density of Arp2/3 branching results in increased contractility.

We do not have an explanation for why NMY-2 increases by twofold following CP depletion. Our FRAP experiments did not reveal a significant difference in NMY-2 dynamics between control and *cap-1* KD conditions, suggesting that the bottleneck for NMY-2 recruitment might be the availability of binding sites on F-actin and that in control conditions F-actin is saturated with NMY-2 such that any increase in F-actin results in a similar increase in NMY-2. Antibody staining against phosphorylated myosin showed that the additional NMY-2 is active, and importantly, laser incision experiments demonstrated that the increase in active NMY-2 is accompanied by an increase in contractility. A role for CP in suppressing actomyosin contractility has recently been shown in cultured fibroblasts (Pocaterra et al., 2019). Ours is the first demonstration of such a role in a living animal.

Based on our Cytosim simulations, it seems that the effects of CAP-1 KD on contractility would be primarily due to the recruitment of myosin. Indeed, the rise in contractility observed *in vivo* could not be attributed solely to the increase of F-actin, given that increasing F-actin on its own, in the simulation, usually led to decreased contractility. Moreover, the contribution of Arp2/3 is always small in our simulations, perhaps as a consequence of our limited exploration of possible architectures. For instance, we only considered actomyosin networks that were isotropically random, because we did not know if the *in vivo* actin could be organized in any particular manner. Based on the Cytosim results, in conjunction with the phenotype of *cap-1* overexpression, the actin network at the rachis bridge is likely a dense network, given that shortening filament length in such networks led to increased contractility, as is also observed experimentally.

Finally, increased contractility is the likeliest cause for the constricted rachis and longer germ cell membrane phenotype in *cap-1(RNAi)* worms. This phenotype has also been observed in worms with *mel-11*/myosin phosphatase depletion. Moreover, a reduction in the myosin activator Rho kinase/LET-502 or in myosin/NMY-2 nullified the *cap-1(RNAi)* effect on rachis constriction, suggesting a role for CP in balancing the contractility of the actomyosin network in the syncytial germline. Higher NMY-2 levels and narrower rachis bridges, resulting from *gck-1* RNAi, have been previously shown to correlate with smaller embryos (Rehain-Bell et al., 2017). This is likely due to reduced cytoplasmic streaming in the rachis, as we have shown for *mel-11* RNAi (Priti et al., 2018). We did not measure embryo size following *cap-1* RNAi, but we observed abnormally large and small oocytes and 50% of the worms were sterile, leading us to conclude that capping protein function is essential for fecundity.

## MATERIALS AND METHODS

### Worm strain maintenance

All *C. elegans* strains were grown and maintained on nematode growth medium (NGM) plates seeded with OP50 bacteria according to standard protocols (Brenner, 1974). All worms were kept at 20°C, unless specified otherwise. All strains used in this study have been listed in Table S1. Some strains in this study were obtained from the Caenorhabditis Genetics Center (CGC; University of Minnesota) and one was made by InVivo Biosystems (Eugene, OR, USA) using CRISPR-Cas9 knock-in.

### CRISPR-Cas9-mediated generation of *cap-1* knockout transgenic worms

For the generation of *cap-1* null mutant, we used the CRISPR-Cas9 method of genome editing (Paix et al., 2017). A 1484-bp deletion was made in the *cap-1* gene using two different sgRNAs. The first guide RNA targeted a PAM site at the end of the first exon (sgRNA1: GCCACCCGGCGAGTTCAACG) and the second guide RNA targeted a PAM site in the 3′ UTR (sgRNA2: TTTGCTTCAAGTTCGTCTGC). A repair template (single-stranded oligodeoxynucleotide; ssODN) was designed flanking 35 nucleotides on each side of the deletion (AAGGTCCGCATTGCTTCGGATTTCATTAAACACGCTGGTCTTCATCATCTTCCTAATCCTCTCCAAAAAC). The injection mix included Cas9 protein (0.8 µg/µl) (Integrated DNA Technologies), *cap-1* sgRNAs (0.06 mM), *cap-1* ssODN (0.225 µg/µl), *dpy-10* sgRNA (0.02 µg/µl), *dpy-10* ssODN (0.04 µg/µl), KCl (25 mM) and *tracr*RNA (0.1 µg/µl). This injection mix was injected into the gonads of young adult stage N2 worms. F1 progeny was screened for phenotypically dumpy worms. These F1 dumpy worms were isolated, allowed to lay embryos, and later screened for *cap-1* deletion by PCR using following primers: forward primer ATCGATGCGCTCATTTCTCT and reverse primer GCGCGACCTTTCTAAAATCA. Worms with the deletion were further confirmed by sequencing. Because *cap-1* mutant worms homozygous for deletion arrested at L2 or L3 larval stage, heterozygous *cap-1* deletion mutants were balanced using a balancer *tmC5* (strain FX30140) (Dejima et al., 2018). This balancer strain has a fluorescent marker, *myo-2p*::Venus, which is expressed strongly in the pharynx, and a recessive uncoordinated phenotype. The balanced *cap-1* deletion heterozygous mutants segregate wild-type GFP-positive heterozygous mutants [*cap-1(−)*/*tmC5*], uncoordinated GFP-positive homozygous balancer worms (*tmC5*/*tmC5)* and non-GFP homozygous *cap-1* deletion mutants [*cap-1(−)*/*cap-1(−)*].

### RNAi

All RNAi experiments were performed by feeding RNAi. Bacterial clones of HT115(DE3) bacterial strain expressing the vector L4440 containing gene specific sequences were obtained from the Ahringer or Vidal libraries (Source BioScience) and sequenced for confirmation. RNAi plates were prepared with NGM media containing 1 mM IPTG and 100 μg/ml of ampicillin. A bacterial clone containing L4440 vector alone was used as a negative control for the experiments, unless specified otherwise. For dsRNA induction, an overnight culture of bacteria was diluted 1:50 and grown at 37°C with shaking for 3-4 h, after which IPTG was added to a final concentration of 1 mM and allowed to grow for an additional 3-4 h. This culture was used to seed the RNAi plates, air-dried and grown overnight at room temperature. Gravid hermaphrodites were bleached on the RNAi plates to release the embryos (unless specified otherwise). Worms hatched on the RNAi plate were grown to adulthood, after which they were collected either for immunofluorescence or live imaging. For mild phenotype, UM208 worms were put on the RNAi plates at L3 stage and grown till adulthood. NMY-2 was depleted partially by growing worms on *L4440(RNAi)+nmy-2(RNAi)* (1:1 ratio) plates from L4 stage until adulthood. For double KD of NMY-2 and CAP-1, worms were grown on *cap-1(RNAi)* plates from hatching until the L4 stage and then transferred to *cap-1(RNAi)+nmy-2(RNAi)* (1:1 ratio) plates until adulthood.

### Phalloidin staining

Gonads were dissected in M9 buffer containing 0.5 mM levamisole on a glass slide. The dissected gonads were fixed in 3.5% formaldehyde in PBS for 20 min, then permeabilized in PBS with 0.025% Triton X-100 for 5 min. Next, the gonads were incubated in phalloidin-TRITC (Sigma-Aldrich, P1951) in the dark at room temperature. The gonads were washed with PBS to remove excess phalloidin and were mounted on a glass slide with Vectashield mounting medium (Vector Laboratories, H-1000) for imaging.

### Immunofluorescence

Dissected gonads (in 0.5 mM levamisole in M9 buffer) were washed to remove levamisole and fixed with 3.5% formaldehyde in PBS for 20 min. Next, the gonads were washed thrice in PBS and then permeabilized with 0.25% Tween 20 in PBS for 10 min. The gonads were then washed thrice with PBS, incubated in blocking solution (1% bovine serum albumin, 0.1% Tween 20 and 30 mM glycine in PBS) at room temperature for 1 h. A 1:400 dilution of anti phosphoMLC Ser19 (Cell Signaling Technology, 3671) (Jenkins et al., 2006) in blocking solution was used to incubate the gonads at 4°C overnight. After three washes with PBS, the gonads were incubated with a solution containing 1:500 anti-rabbit secondary antibody conjugated with Alexa 488 (Invitrogen, A21244), 1:250 phalloidin-TRITC (Sigma-Aldrich, P1951) and 1:1000 DAPI (Sigma-Aldrich) in blocking buffer at room temperature for 1.5 h. After three washes with PBS, the gonads were stored in Vectashield (Vector Laboratories, H-1000) and mounted for imaging.

### Microscopy and image acquisition

Live adult hermaphrodites in 10 mM levamisole or immunostained dissected gonads stored in Vectashield were mounted on fresh 3% agarose pads prepared on a glass slide. Imaging was carried out with a Nikon Ti2E microscope equipped with a Yokogawa W1 spinning-disc system and a Plan Apo 60× oil 1.4 NA and a Plan Apo 100× oil 1.45 NA. Samples were illuminated with 405 nm, 488 nm or 561 nm lasers (Gataca systems) for excitation and acquired on a Prime 95B sCMOS camera (Photometrics). The software MetaMorph version 7.10.2.240 (Molecular Devices) was used as the controlling interface. All images were captured with *z* stacks of 1 μm spacing.

### FRAP

FRAP experiments were performed with the iLAS2 module (Gataca Systems) for targeted laser illumination on the spinning-disc microscope described above with a Plan Apo 100× oil 1.4 NA objective. A circular region of interest (ROI) along the germline rachis of worms expressing NMY-2::GFP was selected manually and photobleached with the 488 nm laser at 80-100% laser power. Images with five *z* slices (1 μm spacing) were acquired using 50% 488 nm laser and exposure time of 400 ms at an interval of 30 s for a total duration of 10 min. The average intensity of the ROI was measured with Fiji for the bleached, unbleached and background ROIs. The fluorescence intensity of the bleached region was double-normalized for photobleaching and background present outside the image using the double-normalization method. The final analysis of difference in recovery rates was calculated with GraphPad Prism 9, as previously described (Priti et al., 2018).

### Laser incisions

An iLAS Pulse system (Gataca Systems) was used for the laser incision experiments. For laser cuts, we used 355 nm laser of ∼16 mW of power pulsed with a frequency of 20 kHz and 0.5 ns pulse width. This laser incision system is attached to a spinning-disc confocal microscope. We immobilized adult worms expressing a membrane marker (mCherry::PLC1δ-PH) and a rachis bridge marker (ANI-2::GFP) using 10 mM levamisole for the control and *cap-1(RNAi)* experiments. Immobilized worms were mounted on a 3% agarose pad, placed on a glass slide, and covered with a coverslip. A small line incision of approximately 10 μm was made at the rachis surface using 80% laser power with ten repetitions and the recoil of the nearby membranes was imaged at a rate of 1 frame per s for a total duration of 1 min. Recoil velocity was calculated by measuring displacement of the germ cell membranes for each time point using the MtrackJ plugin of the Fiji software.

### Image analysis

All image analysis was carried out using Fiji (Schindelin et al., 2012). Rachis straightness was quantified by dividing the length of a straight line between the two ends of the rachis by the length of a path along the centre of the rachis (Fig. 3C). For measurement of germ cell height, several germ cells were selected in the pachytene region in the *z* plane exhibiting the germline midplane. Germ cell height was measured for selected cells (Fig. 3E, 8C, 9C, Fig. S1D,G). Rachis width normalized to gonad width was measured at three distinct regions in the germline midplane, and the average ratio was calculated for each gonad (Fig. 3D,H, 8B, 9B, Fig. S1D,F). We avoided the measurements in the regions where the germ cells were missing in different RNAi conditions and performed measurements only where clear rachis bridges were visible. We quantified rachis bridge perimeter (Fig. 3F) on *z*-projected images using a Fiji macro described previously (Priti et al., 2018).

For fluorescence intensity measurement, each image was corrected for background fluorescence present in the image outside of the worm before measurement. The midplane of the germline rachis was selected for analysis of PLST-1::GFP, CYK-1::GFP, NMY-2::mKate, LifeAct::RFP, and phalloidin at the rachis bridges using Fiji. Mean intensity was measured along a 5-pixel-wide line drawn along the rachis bridges for control, RNAi and overexpression conditions (Figs 3G,H, 5A,B,E,F,I,J, 6A-D, Figs S1C, S3B-D, S7B, S7D,E). For measurement of PLST-1::GFP and phalloidin at the basal and lateral plane, the basal and midplanes of the germline were selected, respectively. The segmented line tool was used to trace the cell boundaries using a 5-pixel-wide line at the basal plane and the cell junctions at the lateral plane and mean intensity was calculated (Fig. 5A,C,D). Normalization of the NMY-2::mKate intensity was determined by calculating the ratio of NMY-2::mKate intensity and membrane marker intensity (GFP::PLC1δ-PH) for each sample (Fig. 6A,B).

Total ARX-2::GFP fluorescence was calculated by tracing the germline outline at the midplane in the meiotic region and mean intensity was measured after subtraction of background fluorescence present in the image outside of the worm (Fig. 5G,H,K).

### Statistical analysis

All statistical analysis was carried out with GraphPad Prism 9 software. Student's *t*-test or one-way analysis of variance (ANOVA) were used to carry out tests of significance for two samples or more than two samples, respectively. All graphs are represented as mean±s.e.m. The sample number for each experiment is indicated in the corresponding figure legend and each experiment was repeated at least three times.

### Cytosim simulations

Cytosim is an Open Source project (gitlab.com/f-nedelec/cytosim) and our results can be reproduced with the configuration file provided in the supplementary Materials and methods. The systems considered in this study are based on those published previously (Belmonte et al., 2017), with the addition of Arp2/3 activity, following methods established by Letort et al. (2015). The simulation was performed in 2D, with bendable filaments connected by three types of bifunctional connectors: crosslinker, Arp2/3 and myosin. The evolution of the system was determined by solving the overdamped Langevin equation because the movement of the filaments is characterized by a low Reynolds number, and by stochastic binding/unbinding of the connectors. Each simulation consisted of two phases. First, the network was created by simulating filaments growing at their barbed/plus ends. These filaments were created by the nucleators, and by branching from Arp2/3 entities. The second phase was started by freezing nucleation, branching, filament growth and by adding motors and crosslinkers. The first stage lasts for 10 s of cytoskeletal time, and second stage 2 s. The size of network *R*(*t*) was calculated during the second stage from the positions of all filaments, and *R*(*t*) was fitted using an exponential *A*.*exp*(−*Bt*) to estimate the contraction rate *B*.

To generate 128 different network architectures, we randomly varied some key parameters of the system, as indicated in Table S2, and then we systematically evaluated the effect of certain perturbations on the contraction rate of the network: having more or less actin, more myosin, more Arp2/3, and combinations of these, as detailed in Table S3. The fixed parameters common to all simulations are detailed in Table S4. Plots were made by comparing the contraction rate of two simulations differing only in one or a few conditions. See supplementary Materials and Methods for further details.

## Supplementary Material

10.1242/develop.201099_sup1 Supplementary information
