# Supplementary material for: Actin-capping protein regulates actomyosin contractility to maintain germline architecture in C. elegans
Source: Development. 2023 Mar 23;150(6):dev201099. doi: 10.1242/dev.201099 (PMC10112912; doi:10.1242/dev.201099)
Supplement: Supplementary information [file develop-150-201099-s1.pdf]

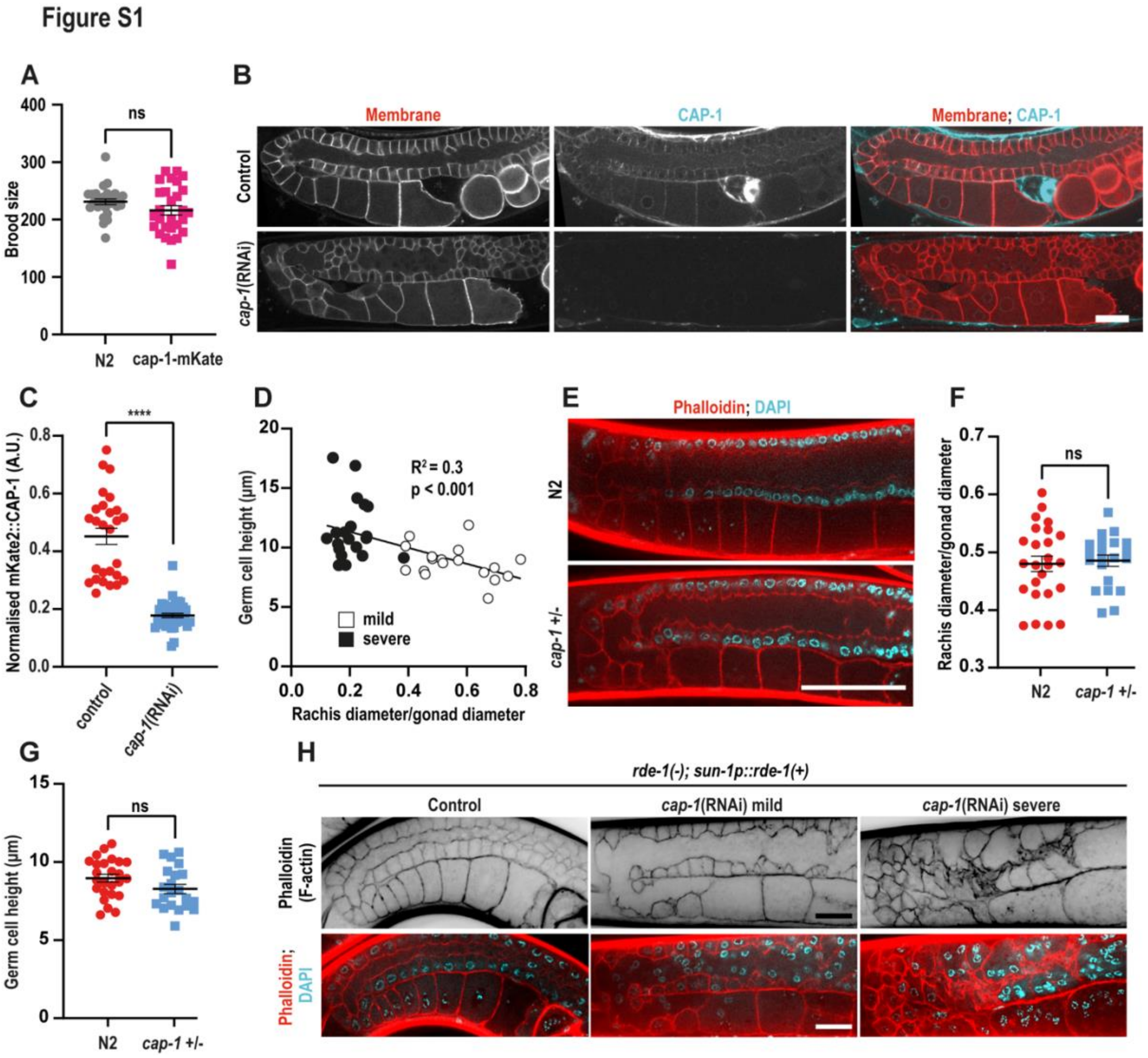

Fig. S1. Endogenously mKate2 tagged CAP-1 expression and depletion

(A) Quantification of brood size in mKate2::CAP-1 worms versus N2 worms. Error bars are ± SEM. ns, non-significant (Student's t-test).

(B) Confocal fluorescence images the *C. elegans* germline expressing GFP::PLC1δ-PH (red) and mKate2::CAP-1 (cyan) in control and *cap-1(RNAi)* worms. Scale bar, 20 µm.

(C) Quantification of normalized mean mKate2::CAP-1 intensity in control (N=27) versus *cap-1(RNAi)* (N=40) worms at the rachis bridge. Error bars are ± SEM. ***p value < 0.001 (Student's t-test). Quantification of fluorescence intensity was measured by drawing a 5-pixel line across the rachis bridges.

(D) Correlation between germ cell height versus rachis width in mildly and severely affected *cap-1(RNAi)* worms.

(E) Midplane confocal images of phalloidin and DAPI stained germlines of N2 and *cap-1* heterozygous mutant. Scale bar, 50 µm

(F-G) Quantification of rachis width and germ cell height in germlines of N2 (n =25) and *cap-1* heterozygous mutants (n= 21).

(H) Top panel- Grey scale phalloidin stained germlines and bottom panel- phalloidin (red) and DAPI (cyan) stained germlines of the *C. elegans* strain deficient in somatic tissue RNAi (*rde-1*(-); *sun-1p::rde-1*(+)) in control and *cap-1(RNAi)* worms. Scale bar, 20 µm.

Figure S2

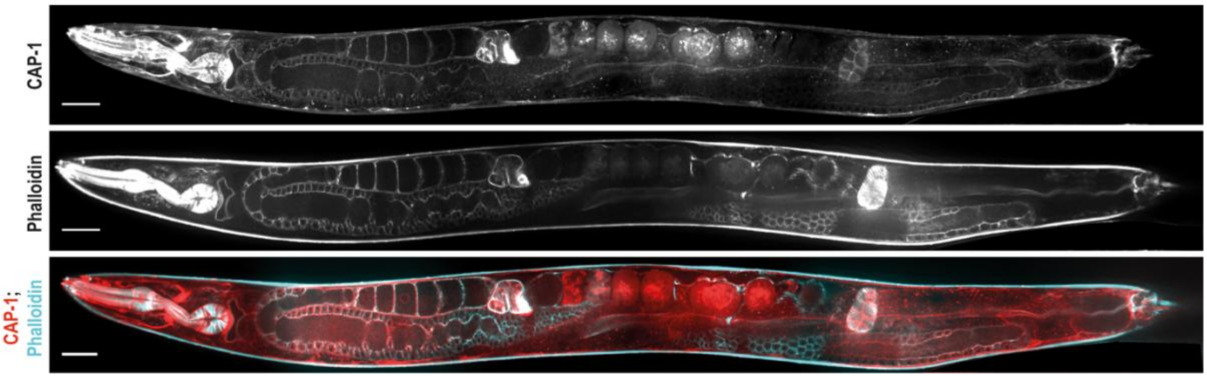

Fig. S2. Phalloidin and CAP-1 localization in the whole worm
Representative confocal fluorescence image of a phalloidin stained (cyan) *C. elegans* hermaphrodite expressing mKate2::CAP-1 (red). Scale bars, 20 µm.

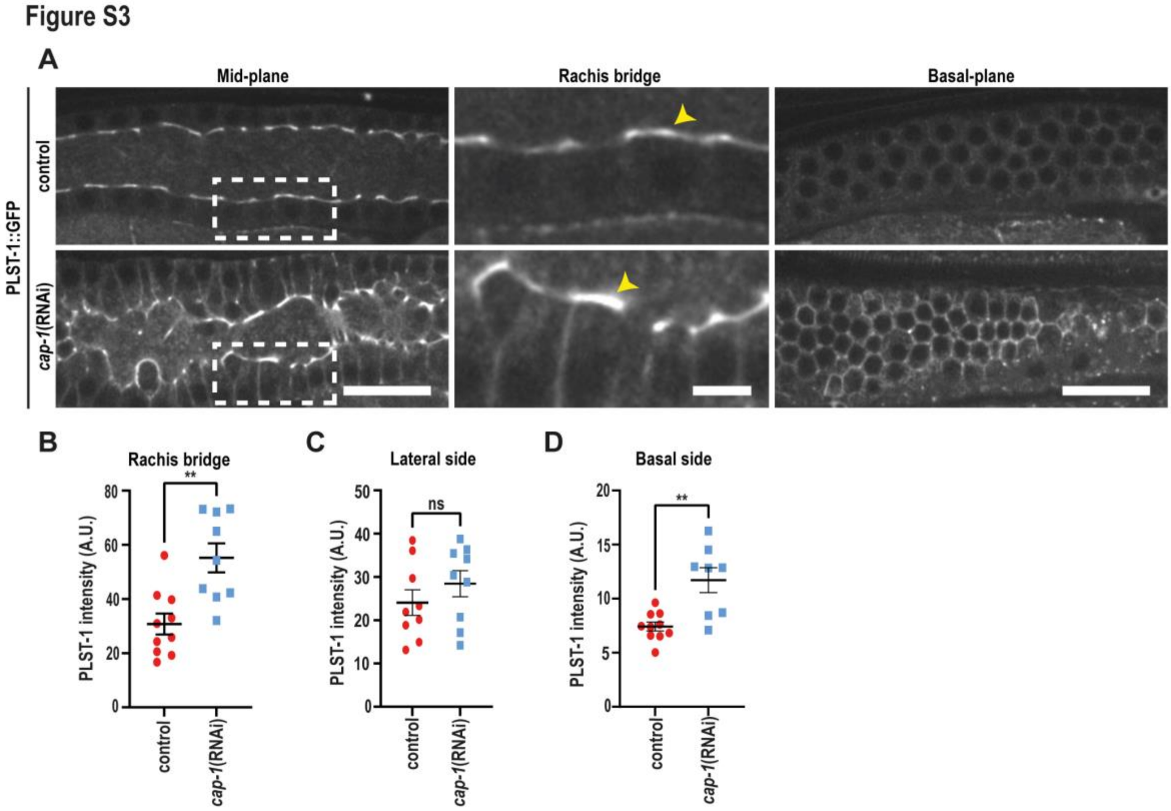

Fig. S3. CAP-1 regulates PLST-1::GFP levels

(A) PLST-1::GFP in control and *cap-1(RNAi)* gonads. Left column shows a mid-plane view; the middle column consists of magnified views of the regions marked by white rectangles in the left column, showing increased intensity of PLST-1::GFP at rachis bridges (arrows). Right column shows basal views of the same germline. Scale bar in left and right columns, 20 µm; scale bar in magnified images, 5 µm.

(B, C, D) Quantification of mean intensity for PLST-1::GFP at rachis bridge, lateral side and basal side of the germ cell membrane in control (N=9) and *cap-1(RNAi)* (N=10) worms. Error bars are ± SEM. ns, non-significant (Student's t-test). Quantification of fluorescence intensity was measured by drawing a 5-pixel line across the rachis bridges.

Development • Supplementary information

Figure S4

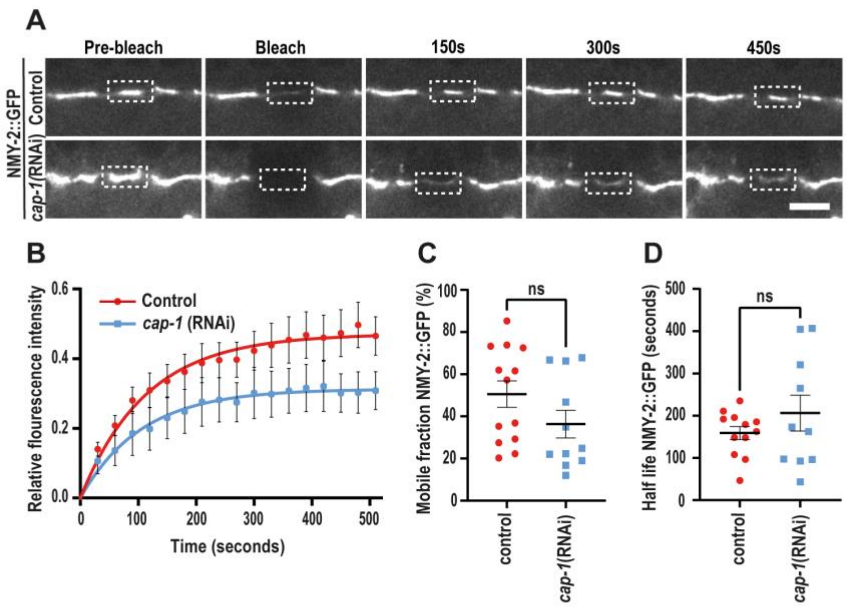

Fig. S4. CAP-1 does not significantly affect the turnover of NMY-2::GFP

(A) Time-lapse confocal images of NMY-2::GFP showing the fluorescence recovery of NMY-2::GFP after photo bleaching at the rachis bridge in control and *cap-1(RNAi)* worms. Scale bar, 5 µm.

(B-D) Quantification of recovery kinetics, mobile fraction, and half-life of NMY-2::GFP (N=11, 13) in control and *cap-1(RNAi)* worms. Error bars are ± SEM. ns, non-significant (Student's t-test).

Development • Supplementary information

Figure S5

A

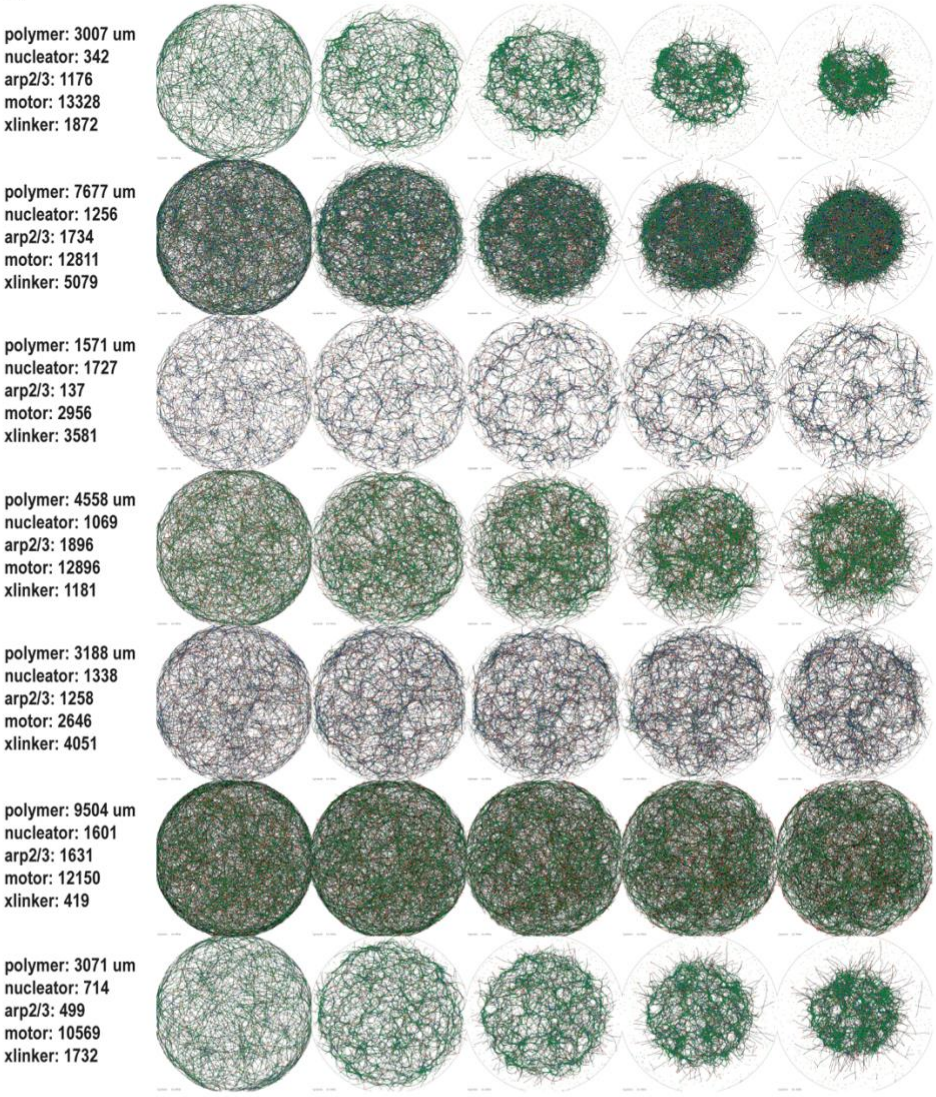

Development • Supplementary information

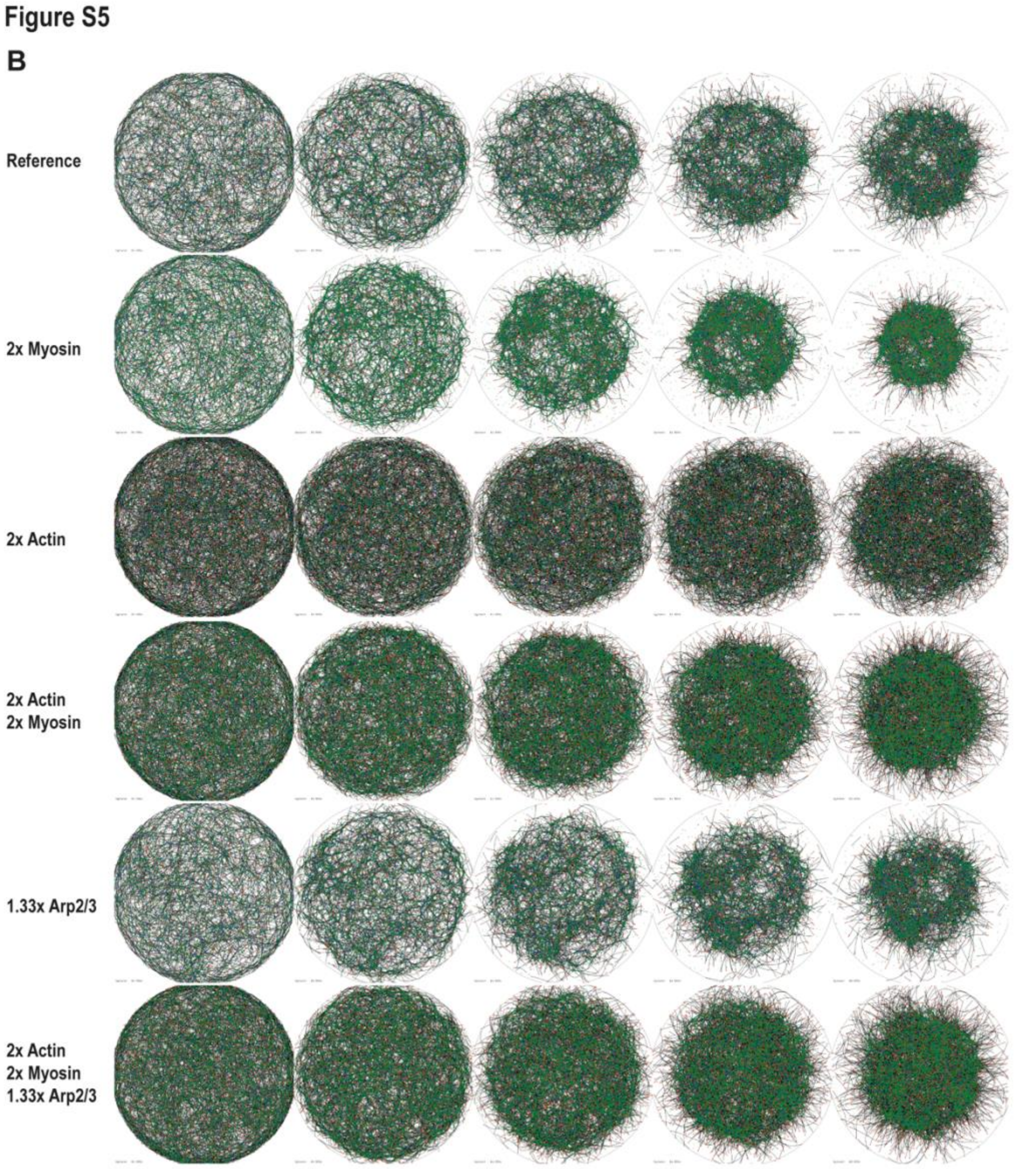

Fig. S5. Examples of randomly generated actomyosin networks and one actomyosin network contraction under varying conditions. (A) Seven examples of randomly generated networks at the end of the actin polymerization phase (Cytosim time 10.00s) and their dynamics over 2.0 Cytosim seconds after the addition of myosin motors and linkers. (B) An example of the dynamics of a single network (one of 128 randomly generated networks) after addition of myosin motors and crosslinkers, in original conditions ('Reference'), and when the original condition was changed as indicated.

Supplementary figure - 6

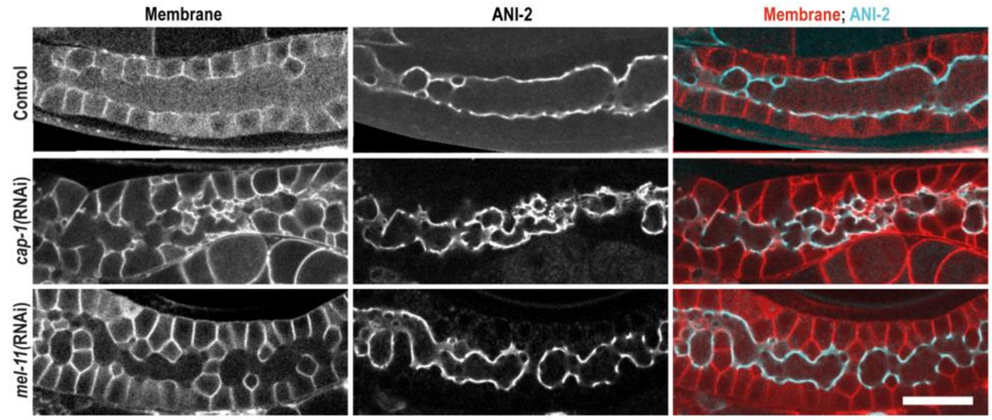

Fig. S6. Rachis constriction in *mel-11* KD and *cap-1* KD worms

Representative confocal fluorescence images of the *C. elegans* germline expressing membrane marker, mCherry::PLC1δ-PH (red) and rachis bridge marker, ANI-2::GFP (cyan) in untreated control, *cap-1(RNAi)* and *mel-11(RNAi)* worms. Scale bar, 20 µm.

Development • Supplementary information

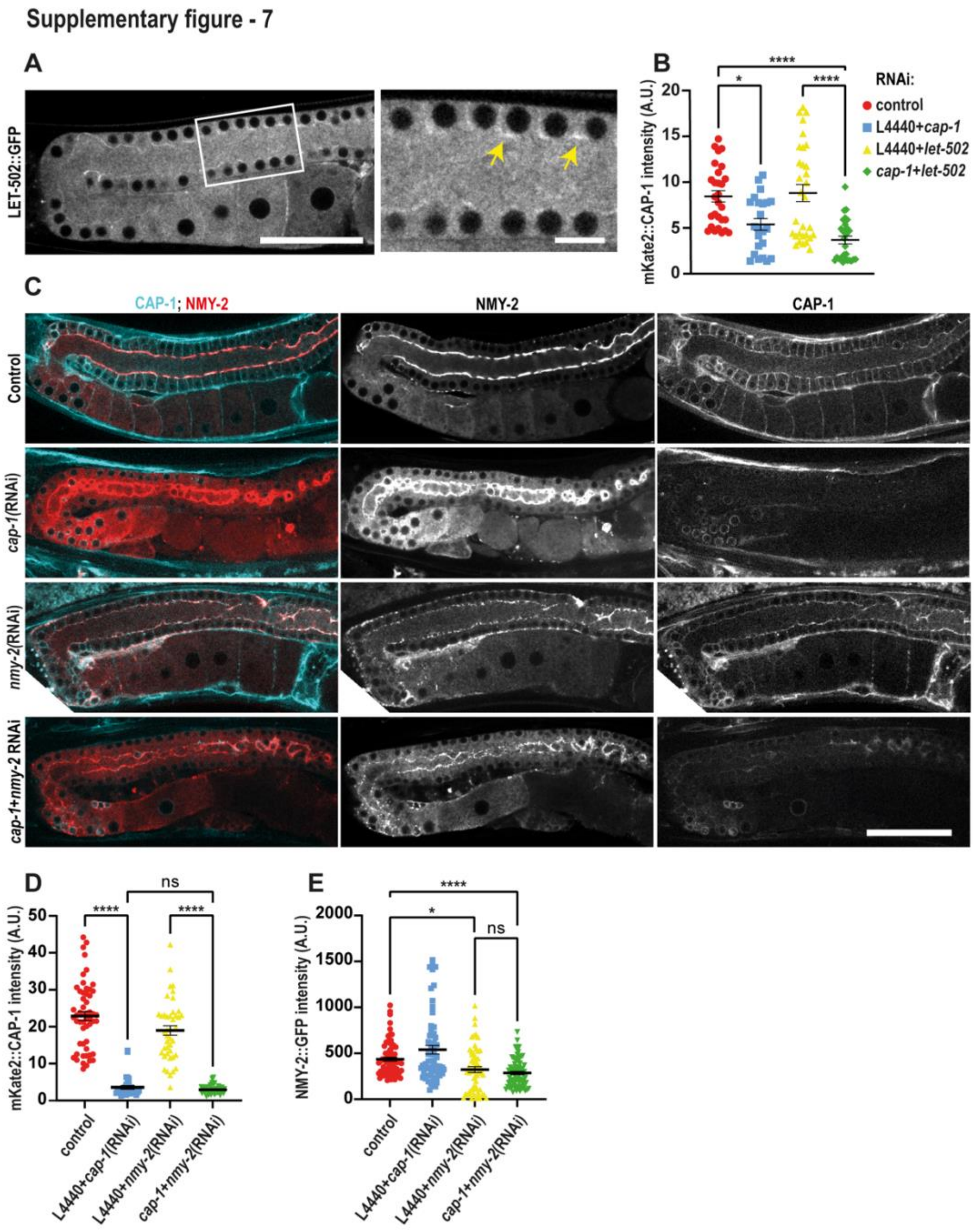

Fig. S7. CAP-1 and NMY-2 intensity in different knock down conditions

(A) Left panel- Confocal fluorescence images of the *C. elegans* germline expressing LET-502 endogenously tagged with eGFP. Right panel- Magnified inset from the left panel show partial enrichment of LET-502::eGFP at the rachis bridges (yellow arrows).

(B) Quantification of CAP-1 intensity at the rachis bridges in control (N=28) , *cap-1(RNAi)* (N=23), *let-502(RNAi)* (N=31) and *cap-1(RNAi)+let-502(RNAi)* (N=28) worms.

(C) Midplane confocal views of the germline expressing mKate2::CAP-1 (cyan) and NMY-2::GFP (red) in control, *cap-1(RNAi)*, *nmy-2(RNAi)* and *cap-1(RNAi)+nmy-2(RNAi)* worms. Scale bar, 50µm.

Development • Supplementary information

(D-E) Quantification of CAP-1 and NMY-2 intensity, respectively, at the rachis bridges in control (N=54) , *cap-1(RNAi)* (N=34), *nmy-2(RNAi)* (N=42) and *cap-1(RNAi)+nmy-2(RNAi)* (N=47) worms.
Quantification of fluorescence intensity was measured by drawing a 5-pixel line across the rachis bridges.

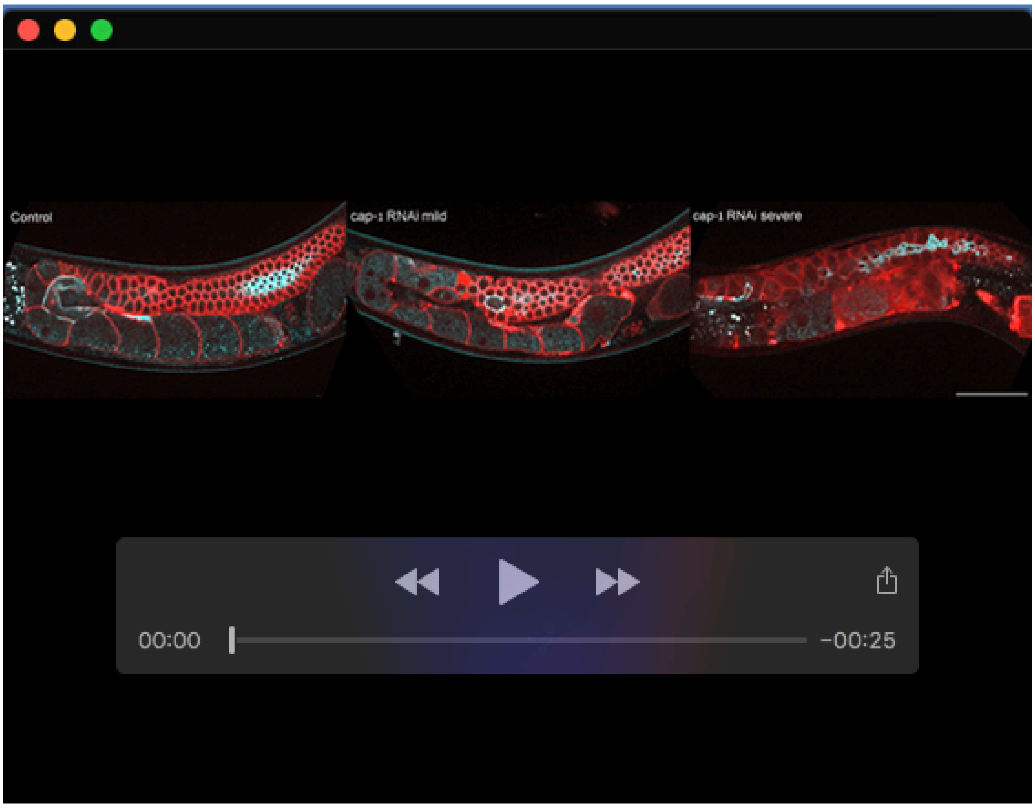

Movie 1. Mildly and severely affected germline of *cap-1*(RNAi) worms. Movie showing the Z-stack images of the germline expressing a membrane marker, mCherry::PLC1δ-PH and a rachis bridge marker, ANI-2::GFP for control (leftmost), mildly (middle) and severely (rightmost) affected *cap-1(RNAi)* worms. Scale bar, 50µm

Development • Supplementary information

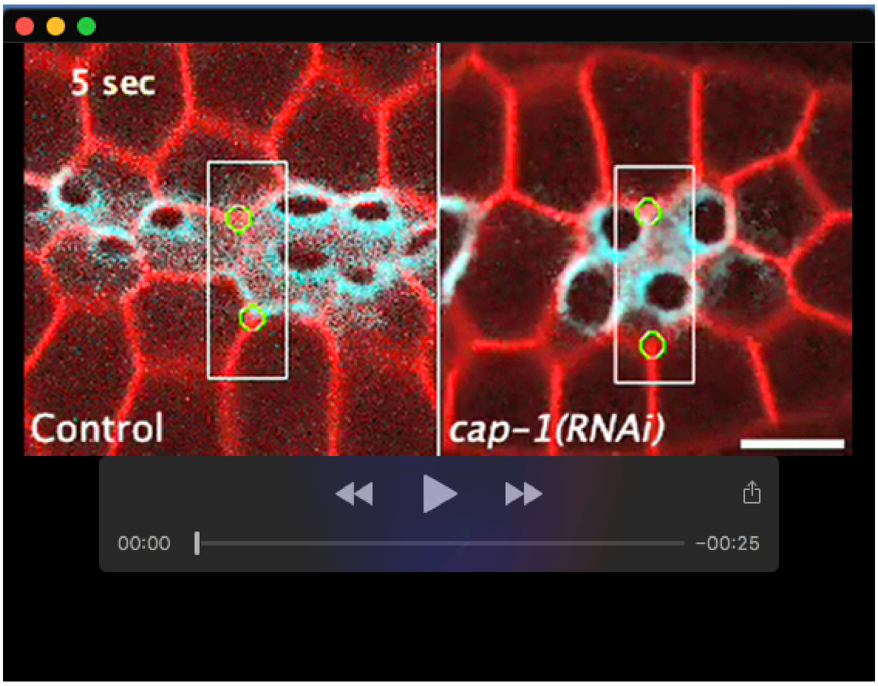

Movie 2. Laser incision in the rachis in control and *cap-1*(RNAi) worms. Time-lapse movie showing the displacement of nearby germ cell vertices (yellow circles) after a laser cut (yellow line) was made at the rachis surface of control and *cap-1*(RNAi) worms expressing GFP::ANI-2 and mCherry::PH. Scale bar, 20 µm

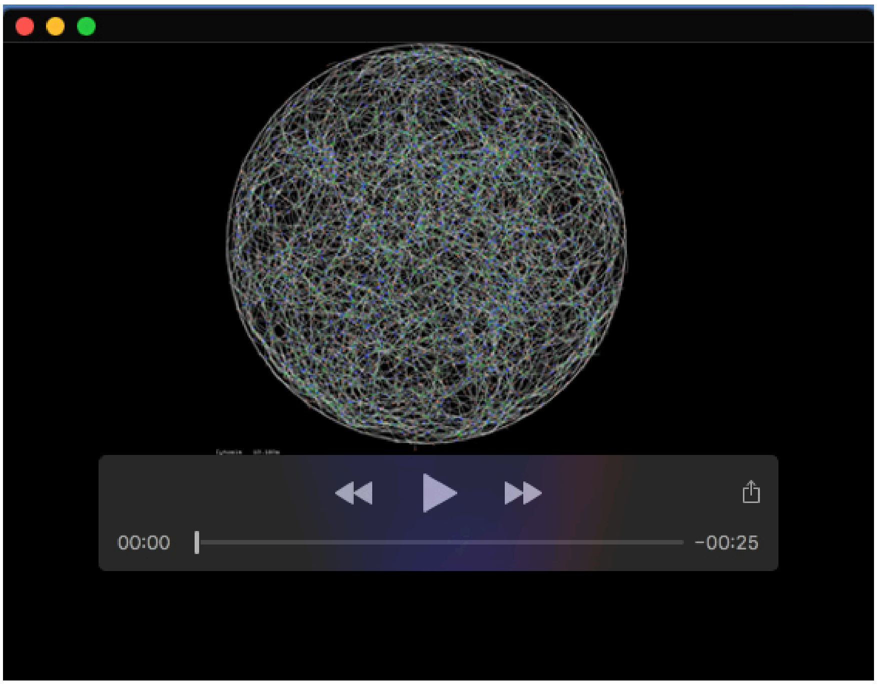

Movie 3. Contraction of simulated actomyosin networks.
The movie shows the same network simulation as depicted on Fig. 5A, in inverted colors. Filaments are painted in white, Myosin in green, Crosslinkers in blue and Arp2/3 in red-orange. The outer circle has a radius of 6µm. The network is composed of 1810 filaments ranging from 0.021 to 4.423µm in length for a total polymer of 4745µm. It contains 3230 crosslinkers, 10280 myosin motors and 315 Arp2/3 entities. The movie covers 2s of time, during which the network shrinks for covering R=6µm to R~3.5µm.

Development • Supplementary information

Supplementary methods

Table S1. List of all strains used

Strain	Genotype	Source
COP1725	*cap-1 (knu650 [pNU1550 - N-terminal degron mKate2 unc-119(+)]) ; unc-119(ed3) III*	InVivo Biosystems
RZB406	*cap-1 (knu650 [pNU1550 - N-terminal degron mKate2 unc-119(+)]) ; unc-119(ed3) III; ltIs38[pAA1; pie-1::GFP::PH(PLC1delta1) + unc-119(+)]*	This study
RZB355	*cap-1 (knu650 [pNU1550 - N-terminal degron mKate2 unc-119(+)]); unc-119(ed3) III; plst-1(msn190[plst-1::gfp]) IV*	This study
RZB347	*cap-1 (knu650 [pNU1550 - N-terminal degron mKate2 unc-119(+)]); cyk-1 (knu83 C-terminal GFP, unc-119 (+)); unc-119(ed3) III*	This study
RZB348	*cap-1 (knu650 [pNU1550 - N-terminal degron mKate2 unc-119(+)]); cas607[arx-2::gfp knock-in] V)*	This study
RZB400	*cap-1 (knu650 [pNU1550 - N-terminal degron mKate2 unc-119(+)] ; nmy-2(cp13[nmy-2::gfp + LoxP]) I.)*	This study
RZB266	*nmy-2 (cp52[nmy-2::mkate]) I;tIs38 [pAA1; pie-1::GFP::PH(PLC1delta1) + unc-119(+)].*	(Priti et al., 2018)
RZB220	*plst-1(msn190[plst-1::gfp]) IV; pie-1:mChe::PH*	This study
LP162	*nmy-2(cp13[nmy-2::gfp + LoxP]) I.*	CGC
UM208	*unc-119(ed3) III; ltIs81 [Ppie-1::gfp-TEV-Stag::ani-2; unc-119 (+)]; ltIs44 [Ppie-1::mCherry::PH(PLC1delta1); unc-119(+)] IV*	(Amini et al., 2014)
OD95	*Itls37 [pAA64; pie-1::mCherry::HIS-58 +unc-119(+). Itls38 [pAA1; pie-1::GFP::PH(PLC1delta1)+unc-119(+)]*	(Essex et al., 2009)
DCL569	*mkcSi13 [sun-1p::rde-1::sun-1 3'UTR + unc-119(+)] II*	(Zou et al., 2019)
RZB282	*LifeAct ::RFP (zbIs2(Ppie-1lifeACT::RFP); nmy-2(cp13[nmy-2::gfp + LoxP]) I.*	(Priti et al., 2018)
FX30140	*tmC5 [F36H1.3(tmIs1220)] IV*	(Dejima et al., 2018)
RZB438	*cap-1 null (msn207) IV/ tmC5*	This study
COP1226	*let-502 (knu278 - C- terminal eGFP loxP)I*	InVivo Biosystems

Details of Cytosim simulations

We only provide here an overview of the methods, since a detailed description is available in the Supplementary Material of Belmonte et al., 2017. In brief, Cytosim uses a Brownian dynamics approach as described previously (Nedelec and Foethke, 2007). Actin filaments are modeled as incompressible bendable filaments with a persistence length of 10 µm in a medium 100 times more viscous than water. They have a fixed length during the contraction phase of the simulation (see below). These filaments are connected by 3 entities: Motors, Crosslinkers and Arp2/3. The evolution of the entire network is simulated by solving the equation of motion for small time intervals, updating this equation as the motor move to different positions on the filaments, and motor and crosslinkers bind or unbind. In essence, the movement is defined by an over-damped Langevin equation: $\xi \frac{dx}{dt} = f(x,t) + B(t)$, for a large multivariate vector x, where the right-hand terms are elastic and random forces respectively, and ξ is a vector of drag coefficients calculated using Stokes' law from the viscosity of the fluid and the dimensions of the objects. Such an equation accurately describes the motion of micrometer-sized objects in a fluid that has the viscosity of cytoplasm. The filaments are elongated objects, discretized as vertices distributed regularly along their length. The differential equation involving all the coordinates of all vertices is solved using a first-order implicit numerical integration scheme. In addition to Brownian motion in each position coordinate, it includes the bending elasticity of the filaments and the elastic terms associated by crosslinkers and motors. All binding and unbinding events are modeled as first-order stochastic processes and we neglected steric interaction between filaments, motors and cross-linkers.

Crosslinkers and Motors were modeled as 2 equivalent units linked in a complex. They diffused in the unbound state, could bind to one or two filaments and, when bound to two filaments, were modeled as Hookean springs of zero resting length and stiffness 250 pN/µm. Binding is determined by a rate within a binding range, and these two parameters are set following typical values for such molecules, initially measured for conventional kinesin. Motor units bind and unbind independently from each other but cannot bind to the same position on the same filament. The unbinding is independent of force and occurs at constant rate. Bound motor units move on their filament with a linear force-velocity relationship characterized by the unloaded speed of 1 µm/s directed towards the plus/barbed end, and a stall force of 4 pN. Motors detach immediately upon reaching the end of the filament, and network contraction is not due to the so called 'end-dwelling' mechanism.

Filaments are created by Nucleators and Arp2/3. A Nucleator/Arp2/3 can create one new filament, of length 0.02µm, that is free to grow at its plus/barbed end while the nucleator stays attached to the minus/pointed end. Thus, the number of filaments in the simulation is always less than the number of Nucleators plus the number of Arp2/3. Apr2/3 was simulated as a complex composed of one Arp2 unit that can bind to filament, and one Arp3 unit with nucleation activity. The Arp3 unit is active only if its partner Arp2 unit is bound. When active, Arp3 can nucleate a new filament, that is then free to grow at its plus/barbed end while the Arp3 stays attached to the minus/pointed end. Moreover, the Arp2/3 complex induces a torque between the two filaments with an equilibrium angle of 72°.

Generation of random network architectures

128 random network architectures were used for Figure 5. To generate these various architecture, key parameters of the system were chosen randomly via uniform sampling within predefined ranges (see table). Particularly, the amount of polymer and the number of Nucleator/Arp23 were varied such as to generate sparse and dense network with various

Development • Supplementary information

qualities. Nucleators and Arp23 were randomly distributed within the circular space and for 10s of actomyosin-time, the filaments were allowed to be nucleated, to grow and move, within the disc. At t=10s the growth of filament was stopped, and motors and crosslinkers were added to the system, distributed randomly onto the crossing formed by filaments. We then simulated the network for 2s of additional time, during which contraction occurred as a consequence of the motor activity and presence of the crosslikers. Examples of random architectures are shown in Supplementary figure 5.

Table S2. Parameters varied to generate random networks

Name	Range of Value	Note
Number of Nucleators	F_0 in [10, 2000]	Each Nucleator/Arp23 can create one filament at most
Number of Arp23	A_0 in [10, 2000]	
Total polymer	$P_0 = (A_0+F_0).L_0$<br>L_0 in [0.5, 3] µm	All filaments share the monomer pool, which limits their growth.
Number of Motors	M_0 in [10, 14000]	
Number of Crosslinkers	X_0 in [10, 7000]	

The quantity P_0 limits the total growth of filaments as follows: each filament growing speed is set dynamically from the total length of the filaments at any given time point i.e.

$$v_g(t) = \alpha\left(1 - \frac{1}{P_0}\sum L_i(t)\right)$$

where $\alpha = 1\mu m/s$ is the maximum growth speed, and $\sum L_i(t)$ is the total length of all filaments in the system at time t. Note that we use a very fast growth speed, because the purpose of growth here is only to generate a random network and not to be realistic. The constant P_0 thus represents an upper bound on the total filament length expressed in length of actin filament ($\sum L_i(t) < P_0$). These assumptions intend to represent conditions in which the amount of G-actin available in the system is finite. As more polymer is made, the growth rate decreases in proportion to the remaining monomer pool. Filaments only grow from the plus/barbed end and all filaments at any time share the same growth speed. Filaments nucleated earlier will thus be longer. Note that P_0 is taken to be proportional to the number of Nucleator + Arp23 in the system. This is a simplifying assumption done to avoid correlations between the number of filaments and their lengths.

Parameters used during network formation

Name	Value	Note
Nucleation rate	0.5/s	Rate of filament birth by Nucleator and Arp23
Growth speed	1µm/s	Maximal growth speed

Contraction rates under different conditions

The radius of the network was measured from the filament's 2D vertex positions x_i:

$$R = \sqrt{\frac{2}{N}\sum_i(\boldsymbol{x}_i - \boldsymbol{c})^2}, \quad \text{with} \quad \boldsymbol{c} = \frac{1}{N}\sum_i \boldsymbol{x}_i \quad \text{the center of mass, for } N \text{ vertices.}$$

The radius $R(t)$ was fitted by a single exponential $A.exp(-Bt)$ to estimate the rate of contraction B, as shown on Fig. 5 E.

Development • Supplementary information

Table S3. Seven conditions simulated for each network architecture. For each network architecture, 7 conditions were simulated

	nucleators	Arp23	polymer	motors	crosslinkers
reference	F_0	A_0	P_0	M_0	X_0
cap knock-out	F_0	$1.3333{\times}A_0$	$2{\times}P_0$	$2{\times}M_0$	X_0
more Arp23	F_0	$1.3333{\times}A_0$	P_0	M_0	X_0
more Actin	F_0	A_0	$2{\times}P_0$	M_0	X_0
more Myosin	F_0	A_0	P_0	$2{\times}M_0$	X_0
more Actin&Myosin	F_0	A_0	$2{\times}P_0$	$2{\times}M_0$	X_0
less Actin	F_0	A_0	$0.66{\times}P_0$	M_0	X_0

Table S4. Fixed parameters used in all simulations. This table lists the parameters of the simulation. Whenever possible, we used published, experimentally determined values. The configuration file of the simulation is also provided as the definitive source of parameter values.

Name	Value	Note
Time step	1 millisecond	Computational parameter. Total time simulated ~ 2s
Viscosity	0.1 pN s/µm²	Effective viscosity of the fluid
k_BT	0.0042 pN µm	Thermal energy at 25°C, defining the Brownian motion of the filaments
Network radius	R = 6 µm	Circular 2D geometry
**Filaments**		
Filament lengths	Varied<br>min: 0.02 µm<br>max: 5 µm	Filaments are found with various length at the end of network formation. The length of all filaments is fixed during the contraction phase.
Filament rigidity	0.05 pN.µm²	F-Actin rigidity (non-stabilized, *in vivo* estimate)
Filament segmentation	0.055 µm	Computational parameter. ~20 actin monomers.
**Motors made of 2 identical subunits**		
Binding	range 20 nm<br>rate 5 s^{-1}	Maximal distance from which a motor can bind to a filament. Rate of binding within this range.
Unbinding	rate 1 s^{-1}	Unbinding is independent of load
Motility	Unloaded speed:<br>$v_m = 1\ \mu m/s$<br>Stall force: $f_s = 4\ pN$	The velocity of a motor varies with force $\vec{f}$, as: $v = v_m\left(1 + \frac{\vec{f}\cdot\vec{d}}{f_s}\right)$, where $\vec{d}$ is the direction in which the motor would move along the filament if it was unloaded.
Link stiffness	$k = 250$ pN/um	Stiffness of the Hookean link between attachment points. If the separation is $\vec{u}$, the force is $\vec{f} = k\vec{u}$
Diffusion constant	100 µm²/s	diffusion rate of unattached motors
**Crosslinkers made of 2 identical subunits**		
Binding	range 20 nm<br>rate 5 s^{-1}	Maximal distance from which a motor can bind to a filament. Rate of binding within this range.
Unbinding	rate 1 s^{-1}	Unbinding is independent of force
Link stiffness	$k = 250$ pN/µm	Stiffness of the Hookean link between attachment

		points. If the separation is $\vec{u}$, the force is $\vec{f} = k\vec{u}$
Diffusion constant	100 μm^2/s	diffusion rate of unattached crosslinkers

Arp2/3, made of Arp2 (binding subunit) and Arp3 (nucleation subunit)

Arp2 binding	range 10 nm<br>rate 5 s^{-1}	Maximal distance from which a motor can bind to a filament. Rate of binding within this range.
Arp2 unbinding	rate 0 s^{-1}	Unbinding does not occur
Link stiffness	$k = 250$ pN/μm	Stiffness of the Hookean link between the side of mother filament and minus end of daughter filament
Link rest angle	$\Theta_0 = 1.22$ rad	Equilibrium angle (72 degrees)
Link angular stiffness	$k_\Theta = 0.5$ pNμm/rad	Stiffness of the Torque link between the side of mother filament and minus end of daughter filament
Diffusion	D = 5 μm²/s	diffusion rate of unattached Arp23

Development • Supplementary information

```
% A branched contractile actin network - FJN 16 Jan 2022
% This is templated configuration file:
% Use preconfig (http://github.com/nedelec/preconfig) to generate Cytosim's configuration files
% Cytosim is an Open Source project (http://gitlab.com/f-nedelec/cytosim)
% It should be compiled in 2D for this simulation

[[F0=random.randint(10,2000)]]
[[A0=random.randint(10,2000)]]
[[P0=int((F0+A0)*random.uniform(0.5, 3))]]
[[M0=random.randint(10,14000)]]
[[X=random.randint(10,7000)]]

[[mod=[0, 1, 2, 4, 5, 7, 8]]]%preconfig.mod=[[mod]]
[[mod1=int(mod&1)]] [[P=int((1+mod1)*P0)]] [[F=int((1+mod1)*F0)]]
[[mod2=int((mod>>1)&1)]] [[A=int((1+0.3333*mod2)*A0)]]
[[mod4=int((mod>>2)&1)]] [[M=int((1+mod4)*M0)]]
[[mod8=int((mod>>3)&1)]] [[K=(1-mod8)*250]]

set simul system
{
    time_step = 0.001
    viscosity = 0.1
    binding_grid_step = 0.1
    dim = 2
}

set space cell
{
    shape = sphere
}

new cell
{
    radius = 6
}

set fiber filament
{
    rigidity = 0.05
    segmentation = 0.22
    confine = inside, 1
    activity = grow
    growing_speed = 1
    total_polymer = [[P]]
}

set hand binder
{
    binding = 5, 0.02
    unbinding = 1, inf
}

set hand plus_motor
{
    binding = 5, 0.02
    unbinding = 1, inf

    activity = move
    unloaded_speed = 1
    stall_force = 4
}

set couple xlinker
{
    hand1 = binder
    hand2 = binder
    stiffness = 250
    diffusion = 100
}

set couple motor
{
    hand1 = plus_motor
    hand2 = plus_motor
    stiffness = 250
    diffusion = 100
}

set hand arp2
{
    binding = 1, 0.01
    unbinding = 0, inf
```


Development • Supplementary information

```
}

set hand arp3
{
    unbinding = 0, inf
    activity = nucleate
    nucleate = 0.5, filament, ( length=0.020; plus_end=grow; )
    nucleation_angle = 1.22
}

set single nucleator
{
    hand = arp3
    diffusion = 5
}

set couple arp23
{
    hand1 = arp2
    hand2 = arp3
    diffusion = 5
    stiffness = [[K]]
    activity = fork
    torque = 0.5, 1.22     % 1.22 radian is 70 degrees
    trans_activated = 1
}

new [[F]] nucleator
new [[A]] arp23

run 8000 system
{
    solve = 0
}
change filament
{
    segmentation = 0.055
}
run 2000 system
change filament
{
    growing_speed = 0, 0
}
change arp3
{
    nucleate = 0
}

new [[M]] motor
new [[X]] xlinker

call equilibrate

run 2000 system
{
    nb_frames = 20}
```
