# Supplementary material for: Actin-capping protein regulates actomyosin contractility to maintain germline architecture in C. elegans
Source: Development. 2023 Mar 23;150(6):dev201099. doi: 10.1242/dev.201099 (PMC10112912; doi:10.1242/dev.201099)
Supplement: Supplementary file 1 [file develop-150-201099_review_history.pdf]

Actin capping protein regulates actomyosin contractility to maintain germline architecture in *C. elegans*

Shinjini Ray, Priti Agarwal, Anat Nitzan, François Nédélec and Ronen Zaidel-Bar
DOI: 10.1242/dev.201099

Editor: Thomas Lecuit

Review timeline
Original submission: 12 July 2022
Editorial decision: 13 September 2022
First revision received: 19 January 2023
Editorial decision: 9 February 2023
Second revision received: 16 February 2023
Accepted: 22 February 2023

Original submission

First decision letter

MS ID#: DEVELOP/2022/201099

MS TITLE: Actin capping protein regulates actomyosin contractility to maintain germline architecture in C. elegans

AUTHORS: Shinjini Ray, Priti Agarwal, Anat Nitzan, Francois Nedelec, and Ronen Zaidel-Bar

i apologise for the long delay before being to come back to you.
I have now received all the referees' reports on the above manuscript, and have reached a decision. The referees' comments are appended below, or you can access them online: please go to BenchPress and click on the 'Manuscripts with Decisions' queue in the Author Area.

As you will see, the referees express considerable interest in your work, but have some criticisms and recommend a revision of your manuscript before we can consider publication. This concerns the knock down approaches using RNAi and quantifications among other things. If you are able to revise the manuscript along the lines suggested, which may involve further experiments, I will be happy receive a revised version of the manuscript. Your revised paper will be re-reviewed by one or more of the original referees, and acceptance of your manuscript will depend on your addressing satisfactorily the reviewers' major concerns. Please also note that Development will normally permit only one round of major revision. If it would be helpful, you are welcome to contact us to discuss your revision in greater detail. Please send us a point-by-point response indicating your plans for addressing the referee’s comments, and we will look over this and provide further guidance.

Please attend to all of the reviewers' comments and ensure that you clearly highlight all changes made in the revised manuscript. Please avoid using 'Tracked changes' in Word files as these are lost in PDF conversion. I should be grateful if you would also provide a point-by-point response detailing how you have dealt with the points raised by the reviewers in the 'Response to Reviewers' box. If you do not agree with any of their criticisms or suggestions please explain clearly why this is so.

© 2023. Published by The Company of Biologists under the terms of the Creative Commons Attribution License (https://creativecommons.org/licenses/by/4.0/).

Reviewer 1

Advance summary and potential significance to field

Ray and coauthors have depleted or overexpressed capping protein and C. elegans to examine its roles in vivo, using the germline to examine roles. A large screening paper from another lab had already reported a germline phenotype for cap-1 depletion. The current manuscript examines the cell biological bases for the phenotype. The authors tagged cap-1 and showed that the protein localizes largely in expected places. They showed quantitatively effects of depletion or overexpression of cap-1 on germline architecture in adults, and that these effects are not seen in larvae after cap-1 knockdown, suggesting a role in maintaining rather than building germline architecture. Cap-1 knockdown results in increased levels of both F-actin and myosin, as well as activated myosin, suggesting multiple possible routes through which cap-1 might affect germline architecture, and laser cuts showed increased tension in the germline rachis. Cytosim simulations suggested increased myosin levels as a driver of this increased tension, and the authors tested this in vivo by depleting a myosin activator along with cap-1, showing that the cap-1 phenotype depends on the myosin activator.

Comments for the author

Strengths of the manuscript are a quantitative, in vivo analysis of complex cytoskeletal contributions to biological form, and a use of experiments to guide modeling, which guided a further experiment that tested a prediction of the modeling. I also view the laser cutting as a strength, to directly show an effect on tension rather than only speculating about forces. I do have some major concerns, though, that I think are important to resolve before the major conclusions of the manuscript can be accepted.

1. My most major concern is that much of the manuscript is based on an RNAi knockdown that resulted in about 60% reduction of protein level and a tagged strain that may have some issues. 60% is only marginally more than would be expected in a cap-1 heterozygote. Do cap-1 heterozygotes have 50% of control protein level, and is there a phenotype? I would expect this to be presented, and if 60% reduction has a phenotype but 50% does not, this should be discussed, at minimum. The tagged protein strain has some peculiarities, for example a strikingly bimodal distribution of protein levels (Supp Fig 1C), and what looks like a much more variable brood size (Supp Fig 1A), and embryo viability is not shown. In Fig 7B, knockdown looks much weaker, and whether knockdown was greater in cap-1 plus let-502 RNAi than in just cap-1 RNAi is not assessed, which makes it impossible to accept the conclusions of the last section of the Results.

2. Data is not shown in several places: embryonic viability on page 2, "40% of the worms with severe germline defects, were sterile and did not produce any embryos" on page 3, "lower penetrance" on page 4,

Other comments
- Given earlier work on capping protein and actin nucleators in vitro, it's not surprising to see complex effects of depleting or adding capping protein, although I think it is an important contribution to show this in vivo. Akin and Mullins 2008 in particular led to clearer thinking about such complex effects. I think it would be useful to introduce this point, and to consider the current results in light of this.
- The past tense of "lead" is "led".
- In some places, constructs like tagged lifeact or tagged PLST-1 are used to make conclusions about F-actin levels, but I could not find information to make clear how quantification was done in a way to get around the issue that these tagged proteins are probably present at the same level in cells with less polymer, only less associated with polymer. I also could not find any details on the lifeact strain.
- page 6, "both known germline actin nucleators": Are no other genes encoding nucleators known to be expressed in the germline? I would be surprised if this is correct.
- Since laser "ablations" traditionally refer to ablating (killing) whole cells, the authors may wish to call them laser cuts instead.
- page 7, "the increase in NMY-2 appears to be mainly due to the increase in F-actin" This seems too speculative for a Results section.

© 2023. Published by The Company of Biologists under the terms of the Creative Commons Attribution License (https://creativecommons.org/licenses/by/4.0/).

- The term "contractility" is, I believe, not a defined and measurable biophysical term but rather is used here to describe imprecisely a few things like tension or contraction rate. The laser cuts assessed tension, and the Cytosim simulations assessed contraction rate, both of which are called contractility currently. I think this needs to be explained more clearly in the manuscript, and the use of contraction rate in the simulations as a proxy for tension needs to be stated and justified in the writing.
- Simulating CP overexpression by reducing the amount of polymer seems too blunt a proxy to me. Why not put capping and formins into the model?

Reviewer 2

Advance summary and potential significance to fieldh2

Ray et al. characterize the role of the actin capping protein CAP-1 in the C. elegans germline gonad by analyzing the localization of the protein and the effects of its depletion. They document that reduced CAP-1 levels lead to an increase in the levels of actin myosin and Arp2/3 complex, that the excess of actin is formin-nucleated and that the germline gonad is under increased tension in these conditions. Their in silico data indicate that the increase in actomyosin contractility is due to increased myosin. Overexpression of the protein is shown to also lead to structural defects in the gonad. The findings extend some evidence already gathered in isolated Capz (mammalian actin capping protein) knockout mouse adult fibroblasts, and in Capz knockout mouse hepatocytes in vivo (Pocaterra et al, 2019), and contribute to the understanding of the regulatory pathways that control actomyosin contractility in vivo. The experiments are in general well done but quantitative methods need to be further explained so that interpretations are clearer and more solid. Additional testing is necessary in some cases and the conclusion that inhibiting actomyosin can prevent increased contractility when capping protein is reduced needs to be better substantiated. Thus, improvements must be made before publication at Development.

Comments for the author

Ray et al. characterize the role of the actin capping protein CAP-1 in the C. elegans germline gonad by analyzing the localization of the protein and the effects of its depletion. They document that reduced CAP-1 levels lead to an increase in the levels of actin, myosin and Arp2/3 complex, that the excess of actin is formin-nucleated and that the germline gonad is under increased tension in these conditions. Their in silico data indicate that the increase in actomyosin contractility is due to increased myosin. Overexpression of the protein is shown to also lead to structural defects in the gonad. The findings extend some evidence already gathered in isolated Capz (mammalian actin capping protein) knockout mouse adult fibroblasts, and in Capz knockout mouse hepatocytes in vivo (Pocaterra et al, 2019), and contribute to the understanding of the regulatory pathways that control actomyosin contractility in vivo. The experiments are in general well done but quantitative methods need to be further explained so that interpretations are clearer and more solid. Additional testing is necessary in some cases and the conclusion that inhibiting actomyosin can prevent increased contractility when capping protein is reduced needs to be better substantiated. Thus, improvements must be made before publication at Development.

Major points:

1 - Overall quantitative analyses need more detailed descriptions in methods and results sections; schematics illustrating how quantifications were done would help:

Figure 2 - Clarify whether quantifications were done in a mix of severe and mild cases. Figure 2B – What were considered abnormal oocytes or what does it mean membrane loss? Insets showing examples would help.
Figure 2F and 7C – how is rachis diameter measured when rachis is convoluted and/or when there seem to be germ cells missing on one side (particularly visible in let-502 RNAi experiments)?
Figure 2H,I, 4B-J, 5B,D, 7B, S1C, S3B-D – how was signal quantified should be explained in greater detail (both in results section and methods)
Fig. 4G – according to panel 4H, the increase in Arx2 signal seems to be mostly cytoplasmic (and not necessarily due to increased signal at the rachis bridge) – how was this accounted for? (this is

© 2023. Published by The Company of Biologists under the terms of the Creative Commons Attribution License (https://creativecommons.org/licenses/by/4.0/).

important to better understand the impact of cyk-1 versus arx2 in the phenotype of cap1 KD and because of the considerations in the computer simulations)

2 - "The decrease in rachis width was accompanied by an increase in germ cell height" As the authors acknowledge, cap-1(RNAi) leads to variable rachis/gonad diameter ratio (Fig. 2F), so this claim can only be done if it is shown that examples with taller germ cells consistently have a decreased rachis width. A graph of rachis width versus germ cell height should be shown in Figure 2.

3 – In a previous publication, the authors show that excessive contractility results in taller germ cells and overly constricted intercellular bridges (Priti et al., 2018). In this manuscript the authors propose that reduced CAP-1 leads to increased contractility, so they should also inspect the perimeter of intercellular bridges.

4 – In the experiments of CAP-1 overexpression clarify whether worms have reached adulthood and are of normal size. The small size of the gonad (narrower gonad) could justify the narrower rachis and the absence of embryos (sterility) could be because the animals are smaller and/or arrested before starting to produce embryos. Also, it seems that that the level of CAP-1 only correlates with rachis width for very narrow rachis (diameter<10μm, Fig. 2H), so the claim that rachis width negatively correlated with CAP-1 levels is overstated. In this experiment, the number of examples should be increased.

5 - The conclusion that CAP-1 is required for gonad maintenance and not gonad formation should be checked in the cap-1 null animals that were generated but not further characterized because they arrested as larva. Depending on the stage at which the larva arrest, it may be possible to conclude whether gonads status is or not in agreement with the larva stage. The RNAi experiment presented in Fig. 3 is not ideal to answer this question, as RNAi treatment duration is different when assessing phenotypes at different larval stages.

6 - Laser ablation experiments are somewhat confusing. The ablation was done at the rachis surface, so a plane of the rachis surface should be shown throughout (the images shown include the germ cells outline, which are more basally located. It is unclear what the "remaining edges" means and where the measurements of the recoil velocity were done. Please clarify.
Also, I could not open movie 1 and its legend is missing.

7 – Computer simulations: what are the nucleators other than arp2/3 that stay bound to the F-actin pointed end (supplemental information and Fig. S5A)? The authors say that the composition of the 128 networks was "chosen randomly". There must have been some kind of logic, no? (the number of each component can vary within a large range, is there really no logic behind the ratios between components in each of the 128 networks analyzed?)

8 – To test whether cap-1 germline defects depend on increased rachis contractility, one important experiment is missing: combine partial depletion of myosin with depletion of CAP1 to reduce the levels of myosin to normal levels. If the hypothesis is correct, the phenotype of cap1 depletion should be rescued. The experiment of rho kinase depletion is not ideal: rho kinase may have other targets other than myosin; its enrichment in the rachis bridges is not shown; depletion probably too drastic (myosin levels should be shown).

9 - Missing (tentative) explanations for: i) the fact that CAP-1 KD leads to increased arp2/3 levels and normal CYK-1 levels, yet CYK-1 contributes to the increased F-actin whereas arp2/3 does not; ii) increased immobile fraction of myosin after CAP-1 KD

Minor points:

- Text should be more carefully written.
- Graph in Fig. 2C, correct y-axis label
- Fig. 7A: panel of cap-1 RNAi does not seem to correspond to the mid section of the gonad
- Fig. S2D – labelling of DNA would help understand whether phenotype is less severe in the germline-specific RNAi gonad
- Fig. S2B – add arrows pointing at what is the spermatheca, the pharynx, the intestine and the

© 2023. Published by The Company of Biologists under the terms of the Creative Commons Attribution License (https://creativecommons.org/licenses/by/4.0/).

germline gonad

- Fig. S3A – remove arrow pointing at increased PLST-1 signal at the lateral, as this is not statistically significant (Fig. S3C). Regarding this, any explanation for actin, but not PLST1, to be increased in the lateral side of the germ cells?
- Fig. S3 title – remove mention to turnover as this is not included in this figure
- Fig. 5E – add labels of fluorescent marker

Reviewer 3

Advance summary and potential significance to field

Despite having been described many decades ago, the *C. elegans* Capping proteins, CAP-1 and CAP-2, have received limited attention. This study by Ray and colleagues directly addresses the role of CAP-1/CAPZA/Capping Protein alpha in the regulation of contractility in the *C. elegans* germline. The Zaidel-Bar lab recently reported a mechanistic model for the architecture of the syncytial gonad of *C. elegans* , and the major role played by carefully balanced actomyosin forces (Priti et al., 2018). This current study adds an important component, by analyzing the localization and role of CAP-1, which is enriched at the rachis bridge structures that are important to maintain the syncytial nature of the germline. Since they cannot use the CRISPR null mutation they generate, which arrests at the larval stage, they use RNAi throughout the paper to test the role of CAP-1, and of its regulators. The RNAi experiments are done with careful controls, and both mild and severe effects are carefully monitored. Altogether, this manuscript makes new discoveries about the role of Capping Protein in germline architecture, including that CAP-1 is required to modulate actomyosin contractility.

The authors show evidence that the germline can form without CAP-1, but the maintenance of its structures, especially the rachis, and therefore the proper formation of oocytes, requires CAP-1. Reduced CAP-1 resulted in a highly constricted rachis, with distorted oocytes, and over-expression of CAP-1 resulted also in a constricted rachis. F-actin is increased at the rachis of *cap-1* RNAi animals. Since the formin CYK-1 is also enriched at the rachis bridges, they test for effects of *cap-1* RNAi on CYK-1:GFP and show CYK-1::GFP can still enrich there. However, loss of *cyk-1* by RNAi blocks the increased F-actin of *cap-1* RNAi, suggesting it is the formins that work with CAP-1 at the rachis bridges. Loss of CAP-1 results in increased NMY-2::GFP, and phospho-MyoII, which fits with the hyper contracted rachis phenotype. To further test if it is increased myosin/NMY-2 activity that leads to *cap-1* defects, they show loss of Rho Kinase (let-502 RNAi) blocks the rachis constrictions in *cap-1* RNAi, and looks like loss of *let-502*. FRAP and laser ablation studies further support the role of CAP-1 in regulating contractility at the rachis. Cytosim simulations to generate 128 architectures by varying parameters including the levels of actin, myosin, cross linkers and Arp2/3, then calculating the contraction rate, supported a model that the actin network is highly dense and that increased myosin would most likely increase contractility, while increased Arp2/3 would not, again supporting that the effects of CAP-1 loss are likely mediated by the effects on increased myosin.

Comments for the author

The manuscript is well written, and the figures are clearly laid out, and well labeled.
I have a few minor suggestions for improvement to this beautiful study.

P. 9 “which everything else kept equal” fix this sentence.
Did you mean, “with everything else kept equal”?

Fig 3A: Can the authors speculate why the rachis ends up discontinuous? Is the idea that excess contractility is ripping the apical structure apart? If so, what adhesive structures are thought to keep this structure together?

Figure 4:E: CYK-1::GFP seems to switch from discontinuous to continuous. Is this always the case, or just due to this particular image? If it is really more continuous, what would this mean regarding the rachis bridges? Are they smaller or fused?

© 2023. Published by The Company of Biologists under the terms of the Creative Commons Attribution License (https://creativecommons.org/licenses/by/4.0/).

4H: ARX-2::GFP in controls and in the cap-1 RNAi animal, the distribution is quite varied along the GL. Is there any pattern to where ARX-2::GFP accumulates, i.e.higher at specific regions of the GL? Describing the control distribution better would help understand the change in the mutant.

Boarder question to better understand the phenotypes: with dysregulated CP, the longer actin fibers could also be floppier, and weaker. Do any of the conditions tested suggest that loss of CAP-1 sometimes creates excess linear actin that destablilizes the structure? I don't have a good suggestion for how to image this. The discontinuous and variable rachis in cap-1 RNAi could be due to areas of excess actomyosin, next to areas of depleted actomyosin. That seems to be what Fig. 5C shows.

One final suggestion: Is there evidence that could be cited that the embryos are larger, smaller or dysregulated in size when myosin and its regulators are altered? That might be a nice correlation to the observations of the shorter or taller germ cell membranes.

First revision

Author response to reviewers' comments

Point by point response to reviewer comments

Reviewer 1 Advance Summary and Potential Significance to Field:
Ray and coauthors have depleted or overexpressed capping protein and C. elegans to examine its roles in vivo, using the germline to examine roles. A large screening paper from another lab had already reported a germline phenotype for cap-1 depletion. The current manuscript examines the cell biological bases for the phenotype. The authors tagged cap-1 and showed that the protein localizes largely in expected places. They showed quantitatively effects of depletion or overexpression of cap-1 on germline architecture in adults, and that these effects are not seen in larvae after cap-1 knockdown, suggesting a role in maintaining rather than building germline architecture. Cap-1 knockdown results in increased levels of both F-actin and myosin, as well as activated myosin, suggesting multiple possible routes through which cap-1 might affect germline architecture, and laser cuts showed increased tension in the germline rachis. Cytosim simulations suggested increased myosin levels as a driver of this increased tension, and the authors tested this in vivo by depleting a myosin activator along with cap-1, showing that the cap-1 phenotype depends on the myosin activator.

Reviewer 1 Comments for the Author:
Strengths of the manuscript are a quantitative, in vivo analysis of complex cytoskeletal contributions to biological form, and a use of experiments to guide modeling, which guided a further experiment that tested a prediction of the modeling. I also view the laser cutting as a strength, to directly show an effect on tension rather than only speculating about forces. I do have some major concerns, though, that I think are important to resolve before the major conclusions of the manuscript can be accepted.

1. My most major concern is that much of the manuscript is based on an RNAi knockdown that resulted in about 60% reduction of protein level and a tagged strain that may have some issues. 60% is only marginally more than would be expected in a cap-1 heterozygote. Do cap-1 heterozygotes have 50% of control protein level, and is there a phenotype? I would expect this to be presented, and if 60% reduction has a phenotype but 50% does not, this should be discussed, at minimum.
Response: While we cannot know the exact protein level in the heterozygous *cap-1+/-* adults, we examined the germline structure in *cap-1+/-* by phalloidin and DAPI staining and compared it to control N2. As shown in a new supplementary figure 1E to 1G, we did not find any defect in the germline of *cap-1+/-* heterozygous adults, suggesting that even if there is a reduction in CAP-1 in the heterozygous (up to 50%) it does not lead to defects. It is important to note that RNAi is somewhat variable and the percentage of KD that we quantified (60% reduction) is an average. We discuss in the results section the requirement for CAP-1 to drop below a threshold in order to show a phenotype in the germline.

© 2023. Published by The Company of Biologists under the terms of the Creative Commons Attribution License (https://creativecommons.org/licenses/by/4.0/).

The tagged protein strain has some peculiarities, for example a strikingly bimodal distribution of protein levels (Supp Fig 1C), and what looks like a much more variable brood size (Supp Fig 1A), and embryo viability is not shown.
Response: We agree with the reviewer that CAP-1 levels in the mKate2:CAP-1 strain show a bimodal distribution pattern. We do not know the reason for it, but we are reassured by the similar brood size (Supplementary Figure 1A) and wild-type level of embryonic viability in this strain, which show that tagging of CAP-1 did not perturb its function. We added to the results section the following quantification of embryonic viability: N2, 99.9% viable embryos, no. of embryos analyzed n= 813; mKate2::CAP-1, 99.4% viable embryos, no. of embryos analyzed n= 716.

In Fig 7B, knockdown looks much weaker, and whether knockdown was greater in cap-1 plus let-502 RNAi than in just cap-1 RNAi is not assessed, which makes it impossible to accept the conclusions of the last section of the Results.
Response: We agree with the reviewer that CAP-1 knockdown was weaker in *cap-1(RNAi)* than in *cap-1(RNAi);let-502(RNAi)* condition. However, this is not a problem but actually it shows that CAP-1 knock down is not diluted when we deplete two genes *i.e. cap-1* and *let-502* simultaneously. Therefore, it supports our conclusion that the narrowing in rachis width and increase in germ cells height observed in *cap-1(RNAi)* are dependent on LET-502, because they are not present in the double KD (as shown in Figure 8 in the revision).
Moreover, to further strengthen the last section of the results, we added in the revision an additional experiment in which we depleted CAP-1 along with NMY-2, and show that the narrowing of the rachis in *cap-1(RNAi)* is also dependent on NMY-2 (Figure 9).

2. Data is not shown in several places: embryonic viability on page 2, "40% of the worms with severe germline defects, were sterile and did not produce any embryos" on page 3, "lower penetrance" on page 4,
Response: We thank the reviewer for pointing out the missing information. As mentioned above, we added the embryonic viability numbers regarding mKate2::CAP-1 to page 2. We quantified the number of worms which showed severe germline defects (as shown in figure 2 and 3, Supplementary movie 1) and failed to produce any progeny after *cap-1* depletion. We found nearly 50% of worm (n=57) failed to lay any progeny and hence, were sterile. We quantified the penetrance of the *cap-1* phenotype in the germline-specific RNAi strain (DCL569), and found it to be 75%. We have included all this information in our revised manuscript.

Other comments
- Given earlier work on capping protein and actin nucleators in vitro, it's not surprising to see complex effects of depleting or adding capping protein, although I think it is an important contribution to show this in vivo. Akin and Mullins 2008 in particular led to clearer thinking about such complex effects. I think it would be useful to introduce this point, and to consider the current results in light of this.
Response: We thank the reviewer for this comment. We added Akin and Mullins, 2008 to the references and added the following sentence to the discussion section: “Complex effects of depleting or adding capping protein were expected based on in vitro studies, which showed that while CP caps elongating actin filaments it also increases the frequency of Arp2/3-mediated nucleation events in a dendritic network and therefore the amount of CP determines the architecture of the actin network (Akin and Mullins, 2008).”

- The past tense of "lead" is "led".
Response: we changed lead to led in the appropriate places.

- In some places, constructs like tagged lifeact or tagged PLST-1 are used to make conclusions about F-actin levels, but I could not find information to make clear how quantification was done in a way to get around the issue that these tagged proteins are probably present at the same level in cells with less polymer, only less associated with polymer. I also could not find any details on the lifeact strain.
Response: First, we’d like to point out that wherever possible we measured F-actin levels from phalloidin stainings. Second, when PLST-1 was measured, we did not make claims on F-actin levels but only on plastin levels. Third, where we used Lifeact-RFP to report on F-actin levels (figure 5J) we quantified the intensity of RFP specifically at the rachis (added to methods). Our assumption is that LifeAct::RFP that is not bound to F-actin is cytosplamic and will not accumulate at the rachis

© 2023. Published by The Company of Biologists under the terms of the Creative Commons Attribution License (https://creativecommons.org/licenses/by/4.0/).

and therefore is not included in our measurements. Finally, we have now included the images of strain expressing LifeAct::RFP in control, *cap-1(RNAi)*, *cap- 1(RNAi);cyk-1(RNAi)*, and *cap-1(RNAi);arx-2 (RNAi)* worms in figure 5I.

- page 6, "both known germline actin nucleators": Are no other genes encoding nucleators known to be expressed in the germline? I would be surprised if this is correct.

Response: The reviewer is correct in pointing out that we did not check all of the known nucleators. Based on the worm tissue expression prediction server developed by the Laboratory for Bioinformatics and Functional Genomics in the Lewis-Sigler Institute for Integrative Genomics at Princeton University, which predicts tissue expression of genes across tissues in *C. elegans* based on microarray and RNA-seq experiments we now can predict that the Ena/VASP ortholog UNC-34 and the formins FRL-1 and INFT-2 are also expressed in the germline. We therefore changed the text from "both known germline nucleators" to "two well known germline nucleators".

- Since laser "ablations" traditionally refer to ablating (killing) whole cells, the authors may wish to call them laser cuts instead.

Response: We agree with the reviewer and have replaced "laser ablation" with "laser incision".

- page 7, "the increase in NMY-2 appears to be mainly due to the increase in F-actin" This seems too speculative for a Results section.

Response: We agree with the reviewer and have deleted this speculative sentence from the results section.

- The term "contractility" is, I believe, not a defined and measurable biophysical term but rather is used here to describe imprecisely a few things like tension or contraction rate. The laser cuts assessed tension, and the Cytosim simulations assessed contraction rate, both of which are called contractility currently. I think this needs to be explained more clearly in the manuscript, and the use of contraction rate in the simulations as a proxy for tension needs to be stated and justified in the writing.

Response: Let us first clarify that the effect of a laser cut is estimated by measuring the contraction rate after severing. We refer to this as 'contractility' and terms like this one (or 'tension') have been widely used in the field to refer to what laser cut tells us, but this is not so simple.

A laser cut severs all connections within the cortex along the cut line and sets tension in the direction orthogonal to the cut line to zero. This results in tension gradients that drive an outward movement of the adjacent cortex away from the cut line. The speed of this retraction is essentially determined by the tension (after the cut) divided by a coefficient of friction that describes frictional interactions between the cortex and its surrounding cytosol and membrane. If the laser has no indirect effects, one can hope that the tension after the cut is close to the tension before the cut, but even in that case it is not possible to determine the tension absolutely (with units of Newtons) because the friction coefficient is unknown. This is explained in simple terms in (Laser ablation to investigate cell and tissue mechanics in vivo, Teresa Zulueta-Coarasa and Rodrigo Fernandez-Gonzalez). The extensive analysis from the Grill group (Determining Physical Properties of the Cell Cortex, Biophysical J. 2016) is based on an active gel theory and considers the shape of the opening and the gradient of retraction speed, but the message is essentially the same. It is possible to determine 3 parameters: the hydrodynamic length, the Maxwell time scale but not the absolute magnitude of the tension. The third parameter that can be determined in principle is a ratio between the "isotropic active stress generated through ATP consumption of myosin" and the "friction coefficient". Thus even with this parameter determined, the absolute tension remains unknown. All other things kept equal, it is expected that faster retraction rates would reflect higher tensions in the cortex, but even this is not certain.

We therefore added the following sentence to the results: "It is not possible to directly measure tension within the actomyosin corset. However, a widely accepted method for assessing tension in the cortex is to measure the speed of retraction following laser incision (Saha et al., 2016). We therefore performed laser cuts at the rachis surface and followed the recoil dynamics of the remaining cell edges (Figure 6E and supplementary movie 2)."

In Cytosim we measured the retraction rate of a crosslinked network with a free boundary. This boundary retracts exactly as if it had been freshly cut from a continuous gel. The retraction speed

© 2023. Published by The Company of Biologists under the terms of the Creative Commons Attribution License (https://creativecommons.org/licenses/by/4.0/).

is essentially equal to the tension generated in the network by the motors, divided by the coefficient of friction of the network. In cytosim this friction term is known as it is the sum of the viscous drag coefficients of all the filaments, calculated from their size and the bulk viscosity. The bulk viscosity is set as estimated in C.elegans cytoplasm (0.1 Pa.s), but this does not help to determine the magnitude of the forces in vivo, because the density of the network and the number of motors are not known.

The geometry of the simulation is different from that of a cut, but a circular isotropic patch gives a very consistent signal that is easy to extract (in contrast to a small cut), and it is faster to compute. We believe that simulating a circular patch of cortex is also more relevant to the unperturbed in vivo situation. In any case the measured quantities in the experiments (recoil velocity) and in the simulation (retraction velocity) are essentially the same proxy for force, given the pitfall that the friction cannot be determined.

To clarify this point we added the following text to the description of the simulation: "Although their geometry is different, the retraction of a patch of cortex in the simulation is analogous to the retraction of the cortex following an experimental laser incision. One can imagine that the onset of the simulation is immediately after a circular laser cut extracted the cortical patch from the larger cortex.".

- Simulating CP overexpression by reducing the amount of polymer seems too blunt a proxy to me. Why not put capping and formins into the model?
Response: We agree that it would have been possible indeed to add capping molecules into the simulation, and in fact anyone wishing to do this will be able to follow up on our work, since we provide all necessary material: source code, configurations, for this. This is indeed maybe a crude approximation but we wanted to keep the model simple, and this had the virtue of not adding unknown parameters to the problem. From the experiments we could see that the quantity of polymer was reduced and we thus simply implemented this observation. Adding the number of capping/forming and associated binding/unbinding rate would have forced us to fit/guess/vary these rates.

Reviewer 2 Comments for the Author:
Ray et al. characterize the role of the actin capping protein CAP-1 in the C. elegans germline gonad by analyzing the localization of the protein and the effects of its depletion. They document that reduced CAP-1 levels lead to an increase in the levels of actin, myosin and Arp2/3 complex, that the excess of actin is formin-nucleated and that the germline gonad is under increased tension in these conditions. Their in silico data indicate that the increase in actomyosin contractility is due to increased myosin. Overexpression of the protein is shown to also lead to structural defects in the gonad. The findings extend some evidence already gathered in isolated Capz (mammalian actin capping protein) knockout mouse adult fibroblasts, and in Capz knockout mouse hepatocytes in vivo (Pocaterra et al, 2019), and contribute to the understanding of the regulatory pathways that control actomyosin contractility in vivo. The experiments are in general well done but quantitative methods need to be further explained so that interpretations are clearer and more solid. Additional testing is necessary in some cases and the conclusion that inhibiting actomyosin can prevent increased contractility when capping protein is reduced needs to be better substantiated. Thus, improvements must be made before publication at Development.

Major points:
1 - Overall quantitative analyses need more detailed descriptions in methods and results sections; schematics illustrating how quantifications were done would help:
Figure 2 - Clarify whether quantifications were done in a mix of severe and mild cases.
Response: Quantifications were carried out on *cap-1(*RNAi) germlines with mild as well as severe phenotypes. This was explained in the text: "Quantification of the frequency of appearance of these phenotypes in the entire cap-1(RNAi) population is shown in figure 2C and 2D." and now also in the figure legend: "(C) Percentage of gonads showing the occurrence of the phenotypes 'abnormal oocytes', 'nuclei in rachis' and 'membrane loss' in cap-1(RNAi) worms (with either mild and severe phenotype).".

Figure 2B – What were considered abnormal oocytes or what does it mean membrane loss? Insets showing examples would help.

© 2023. Published by The Company of Biologists under the terms of the Creative Commons Attribution License (https://creativecommons.org/licenses/by/4.0/).

Response: As suggested by the reviewer, we have included large magnified insets in our revised figure 2B displaying regions of membrane loss (pink box), nuclei mispositioned within the rachis (green box and green arrowhead), multinucleated germ cells (yellow box and yellow arrowhead), and abnormal oocyte with multiple nuclei (blue box and blue arrowheads) in *cap- 1*(RNAi).

Figure 2F and 7C – how is rachis diameter measured when rachis is convoluted and/or when there seem to be germ cells missing on one side (particularly visible in let-502 RNAi experiments)?
Response: We measured the rachis width at three distinct meiotic regions (before the loop region) in the gonads which were not highly convoluted. We avoided the measurements in the regions where the germ cells were missing in different RNAi conditions and performed measurements only where clear rachis bridges were visible.

Figure 2H,I, 4B-J, 5B,D, 7B, S1C, S3B-D – how was signal quantified should be explained in greater detail (both in results section and methods)
Response: As suggested by the reviewer, we have detailed for each figure the method of quantification in the revised Methods and Figure legends.

Fig. 4G – according to panel 4H, the increase in Arx2 signal seems to be mostly cytoplasmic (and not necessarily due to increased signal at the rachis bridge) – how was this accounted for? (this is important to better understand the impact of cyk-1 versus arx2 in the phenotype of cap1 KD and because of the considerations in the computer simulations)
Response: Since ARX-2::GFP has high cytoplasmic localization and no significant enrichment at the rachis bridges, as the reviewer pointed out, we did not measure ARX-2::GFP intensity specifically in the rachis bridges. Rather, ARX-2::GFP fluorescence was calculated by tracing the entire meiotic region of the germline at the midplane and the mean intensity was plotted.

2 - "The decrease in rachis width was accompanied by an increase in germ cell height" As the authors acknowledge, cap-1(RNAi) leads to variable rachis/gonad diameter ratio (Fig. 2F), so this claim can only be done if it is shown that examples with taller germ cells consistently have a decreased rachis width. A graph of rachis width versus germ cell height should be shown in Figure
Response: As suggested by the reviewer, we compared the rachis width normalized to gonad width with the germ cell height of *cap-1(RNAi)* worms and found that the gonads with narrower rachis have longer germ cells. We have included a new graph showing the negative correlation between rachis width and germ cell height of *cap-1(RNAi)* worms in Supplementary figure 1D.

3 – In a previous publication, the authors show that excessive contractility results in taller germ cells and overly constricted intercellular bridges (Priti et al., 2018). In this manuscript the authors propose that reduced CAP-1 leads to increased contractility, so they should also inspect the perimeter of intercellular bridges.
Response: We thank the reviewer for suggesting this quantification. We quantified the perimeter of rachis bridges in control versus *cap-1(RNAi)* gonads and display these new results as a graph in Figure 3F. This analysis revealed that rachis bridge perimeter is significantly reduced in *cap-1(RNAi)* worms, as expected.

4 – In the experiments of CAP-1 overexpression clarify whether worms have reached adulthood and are of normal size. The small size of the gonad (narrower gonad) could justify the narrower rachis and the absence of embryos (sterility) could be because the animals are smaller and/or arrested before starting to produce embryos. Also, it seems that that the level of CAP-1 only correlates with rachis width for very narrow rachis (diameter<10micron, Fig. 2H), so the claim that rachis width negatively correlated with CAP-1 levels is overstated. In this experiment, the number of examples should be increased.
Response: We agree with the reviewer that some of the gonads overexpressing CAP-1 were small and sterile, and hence the decrease in rachis width could be due to smaller gonads. To rule out this possibility, we normalized the rachis width with the gonad width of each of the worms analyzed and compared it with the mKate2::CAP-1 intensity. We found a strong negative correlation between mKate2::CAP-1 intensity and rachis width with a p value <0.0001 and a correlation coefficient R^2= 0.78 (Figure 3H) suggesting that CAP-1 overexpressing worms have narrower rachis. Also, as suggested by the reviewer we repeated the experiment several more times to increase the number of gonads analyzed (n=13) for CAP-1 overexpression (previously, n= 6).

© 2023. Published by The Company of Biologists under the terms of the Creative Commons Attribution License (https://creativecommons.org/licenses/by/4.0/).

5 - The conclusion that CAP-1 is required for gonad maintenance and not gonad formation should be checked in the cap-1 null animals that were generated but not further characterized because they arrested as larva. Depending on the stage at which the larva arrest, it may be possible to conclude whether gonads status is or not in agreement with the larva stage. The RNAi experiment presented in Fig. 3 is not ideal to answer this question, as RNAi treatment duration is different when assessing phenotypes at different larval stages.
Response: We thank the reviewer for suggesting this experiment. To rule out the possibility of inefficient knockdown in the early larval stages, we analyzed the gonads in *cap-1* null mutants. Phalloidin staining showed that gonad formation was unaffected in the arrested larvae of the *cap-1* null mutants (Figure 4C), which were identified by not having the balancer (non-green) and being arrested as larvae.

6 - Laser ablation experiments are somewhat confusing. The ablation was done at the rachis surface, so a plane of the rachis surface should be shown throughout (the images shown include the germ cells outline, which are more basally located. It is unclear what the "remaining edges" means and where the measurements of the recoil velocity were done. Please clarify. Also, I could not open movie 1 and its legend is missing.
Response: We carried out the laser ablation experiments on the rachis surface and calculated the recoil velocity by the measuring the displacement of two nearby vertices of the germ cell membranes (as displayed in our revised figure 6E and Supplementary Movie 2). The reasons we carried out the analysis in this way are: (1) after laser ablation the intensity of ANI-2::GFP, a marker for rachis bridges, bleaches out rapidly and hence, the rachis surface is not visible throughout (2) absence of any specific vertices at the rachis surface makes it difficult to trace the displacement with time. We have made sure that movie is in the correct format now and could be opened. Also, we have included the legend for the movie in the Supplementary file.

7 – Computer simulations: what are the nucleators other than arp2/3 that stay bound to the F-actin pointed end (supplemental information and Fig. S5A)?
Response: We use a nucleator that stays attached to the pointed end (Arp3), but this entity does nothing more than nucleating. In the simulation this molecule has no drag coefficient and is not a crosslinker, and thus it does not influence the position of the filament, after the nucleation event. With a nucleator that would stay attached to the barbed end, the position of the nucleator would be changed by filament assembly, and this could affect to position where other filaments would be nucleated, but since in our setup there is no filament turnover, the position of the nucleator is irrelevant. One nucleator nucleates only one filament. Hence the results of the simulation would have been identical. Note that this would not be true if the nucleator had been immobilized on a pattern. The only thing that matter in our case is where these filaments were nucleated, and this occurred uniformly.

The authors say that the composition of the 128 networks was "chosen randomly". There must have been some kind of logic, no? (the number of each component can vary within a large range, is there really no logic behind the ratios between components in each of the 128 networks analyzed?)
Response: Unfortunately, the characteristics of the actomysin network invivo are unknown. Our choice was either to guess the values, or to probe many possibilities and see what happens. We naturally preferred the later approach. For the exploration we resorted to random sampling, within ranges that are defined widely, and guided by our expertise of these system: for example we assumed that it would not be generally useful to probe networks that have way more than 7 motors than filaments. And thus the range of motor is up to 14000, when the range of filament is up to 2000. It turned out that the results were quite consistent (despite the wild range of parameters, we could make sense of the outcome), and we thus decided to keep the whole range of simulations.

The number of nucleators is a random integer F in [10, 2000].
The number of Arp23 is a random integer A in [10, 2000]
The total length of polymer available is (A+F) multiplied by a random number in [0.5, 3]
The number of myosin motor is a random integer in [10, 14000]
the number of crosslinkers is a random integer in [10, 7000]

All parameters are set independently, except for the total polymer length available.

© 2023. Published by The Company of Biologists under the terms of the Creative Commons Attribution License (https://creativecommons.org/licenses/by/4.0/).

8 – To test whether cap-1 germline defects depend on increased rachis contractility, one important experiment is missing: combine partial depletion of myosin with depletion of CAP1 to reduce the levels of myosin to normal levels. If the hypothesis is correct, the phenotype of cap1 depletion should be rescued. The experiment of rho kinase depletion is not ideal: rho kinase may have other targets other than myosin; its enrichment in the rachis bridges is not shown; depletion probably too drastic (myosin levels should be shown).
Response: We thank the reviewer for suggesting these experiments. To confirm that the germline defects observed in *cap-1(RNAi)* worms were due to increased contractility, we carried out partial depletion of NMY-2 in *cap-1*(RNAi) gonads, as suggested by the reviewer. Gonads depleted of both NMY-2 and CAP-1 had a wider rachis and shorter germ cells, phenocopying partial depletion of NMY-2, suggesting that the hypercontractile rachis phenotype of *cap- 1*(RNAi) gonads is dependent on NMY-2 (Figure 9 and Supplementary figure 7C-E).
We retained the data on LET-502;CAP-1 double knockdown and in addition included a new figure displaying the localization of LET-502::GFP. We saw a partial enrichment of LET- 502::GFP at rachis bridges (yellow arrows, Supplementary figure 7A).

9 - Missing (tentative) explanations for: i) the fact that CAP-1 KD leads to increased arp2/3 levels and normal CYK-1 levels, yet CYK-1 contributes to the increased F-actin whereas arp2/3 does not; ii) increased immobile fraction of myosin after CAP-1 KD
Response: We do not have an explanation for why CAP-1 KD increases arp2/3 and not cyk-1, but we did add to the discussion a (tentative) explanation for why cyk-1 contributes to increased F-actin and arp2/3 does not. The exact text is as follows: "Interestingly, we found this increase in F-actin to be entirely dependent on the formin CYK-1 and independent of the Arp2/3 complex, despite the fact that Arp2/3 levels increased upon cap-1(RNAi) while CYK-1 levels remained constant. These findings suggest that the actin cytoskeleton at the rachis is primarily polymerized by CYK-1, a conclusion that is consistent with the prominent localization of CYK-1 at rachis bridges. Arp2/3, in contrast, is not enriched at the rachis, and so the increase in cytoplasmic Arp2/3 following cap-1 depletion might have an effect on cytoplasmic actin networks in the germline, which were beyond the scope of this study.".
As for the increase in immobile fraction of myosin after CAP-1 KD, this was not statistically significant and therefore we prefer not to speculate on its cause.

Minor points:
- Text should be more carefully written.
- Graph in Fig. 2C, correct y-axis label

Response: We have corrected the label in our revised figure (Figure 2D).

- Fig. 7A: panel of cap-1 RNAi does not seem to correspond to the mid section of the gonad

Response: We thank the reviewer for pointing it out. We have replaced the *cap-1(RNAi)* image with a better image where the mid-section of the gonad is clearly visible (Figure 8A).

- Fig. S2D – labelling of DNA would help understand whether phenotype is less severe in the germline-specific RNAi gonad

Response: As suggested by the reviewer, we have included a new panel to the Supplementary figure 1H (previously Supplementary figure 2D) displaying the images of control and germline- specific *cap-1(RNAi)* gonads stained with phalloidin and DAPI, a DNA marker.

- Fig. S2B – add arrows pointing at what is the spermatheca, the pharynx, the intestine and the germline gonad

Response: We have marked the corresponding regions and displayed some of them as magnified images in Figure 1A.

- Fig. S3A – remove arrow pointing at increased PLST-1 signal at the lateral, as this is not statistically significant (Fig. S3C). Regarding this, any explanation for actin, but not PLST1, to be increased in the lateral side of the germ cells?

Response: As suggested by the reviewer, we have removed the arrow pointing at PLST-1 signal at the lateral side of the germ cell. We don't know the reason we see an increase in actin intensity but not PLST-1 intensity at lateral germ cell membrane. One possible explanation is that PLST-1 has a preference for linear CYK-1-polymerized F-actin, which is enriched at the rachis, whereas the cortex on the lateral sides of germ cells is enriched with arp2/3-polymerized F-actin.

© 2023. Published by The Company of Biologists under the terms of the Creative Commons Attribution License (https://creativecommons.org/licenses/by/4.0/).

- Fig. S3 title – remove mention to turnover as this is not included in this figure

Response: We thank the reviewer for pointing this error in the title and we have removed the text as suggested.

- Fig. 5E – add labels of fluorescent marker

Response: We have included the labelling of the fluorescent markers in our revised figure (Figure 6E).

Reviewer 3 Advance Summary and Potential Significance to Field:
Despite having been described many decades ago, the *C. elegans* Capping proteins, CAP-1 and CAP-2, have received limited attention. This study by Ray and colleagues directly addresses the role of CAP-1/CAPZA/Capping Protein alpha in the regulation of contractility in the *C. elegans* germline. The Zaidel-Bar lab recently reported a mechanistic model for the architecture of the syncytial gonad of *C. elegans* , and the major role played by carefully balanced actomyosin forces (Priti et al., 2018). This current study adds an important component, by analyzing the localization and role of CAP-1, which is enriched at the rachis bridge structures that are important to maintain the syncytial nature of the germline. Since they cannot use the CRISPR null mutation they generate, which arrests at the larval stage, they use RNAi throughout the paper to test the role of CAP-1, and of its regulators. The RNAi experiments are done with careful controls, and both mild and severe effects are carefully monitored. Altogether, this manuscript makes new discoveries about the role of Capping Protein in germline architecture, including that CAP-1 is required to modulate actomyosin contractility.

The authors show evidence that the germline can form without CAP-1, but the maintenance of its structures, especially the rachis, and therefore the proper formation of oocytes, requires CAP-1. Reduced CAP-1 resulted in a highly constricted rachis, with distorted oocytes, and over-expression of CAP-1 resulted also in a constricted rachis. F-actin is increased at the rachis of *cap-1* RNAi animals. Since the formin CYK-1 is also enriched at the rachis bridges, they test for effects of *cap-1* RNAi on CYK-1:GFP and show CYK-1::GFP can still enrich there. However, loss of *cyk-1* by RNAi blocks the increased F-actin of *cap-1* RNAi, suggesting it is the formins that work with CAP-1 at the rachis bridges. Loss of CAP-1 results in increased NMY-2::GFP, and phospho-MyoII, which fits with the hyper contracted rachis phenotype. To further test if it is increased myosin/NMY-2 activity that leads to *cap-1* defects, they show loss of Rho Kinase (let-502 RNAi) blocks the rachis constrictions in *cap-1* RNAi, and looks like loss of *let-502*. FRAP and laser ablation studies further support the role of CAP-1 in regulating contractility at the rachis. Cytosim simulations to generate 128 architectures by varying parameters including the levels of actin, myosin, cross linkers and Arp2/3, then calculating the contraction rate, supported a model that the actin network is highly dense and that increased myosin would most likely increase contractility, while increased Arp2/3 would not, again supporting that the effects of CAP-1 loss are likely mediated by the effects on increased myosin.

Reviewer 3 Comments for the Author:
The manuscript is well written, and the figures are clearly laid out, and well labeled.
I have a few minor suggestions for improvement to this beautiful study.

P. 9 “which everything else kept equal” fix this sentence.
Did you mean, “with everything else kept equal”?
Response: We changed the wording of the sentence so it wouldn’t be confusing. Instead of saying “with everything else kept equal” we used “increasing F-actin on its own”.
The new sentence reads: “Indeed, the raise in contractility observed in vivo could not be attributed solely to the increase of F-actin, since increasing F-actin on its own, in the simulation, usually led to decreased contractility.”

Fig 3A: Can the authors speculate why the rachis ends up discontinuous? Is the idea that excess contractility is ripping the apical structure apart? If so, what adhesive structures are thought to keep this structure together?
Response: The rachis in *cap-1(RNAi)* never ends up discontinuous. The rachis looks like it is discontinuous in what was figure 3A and now is figure 4B because the image is a single focal plane

© 2023. Published by The Company of Biologists under the terms of the Creative Commons Attribution License (https://creativecommons.org/licenses/by/4.0/).

and the rachis is meandering in and out of the focal plane. The high degree of meandering of *cap-1(RNAi)* is quantified in figure 3C ("rachis straightness") and in figure 3B, which shows a rachis with a maximum intensity Z-projection, it is possible to see that while it meanders, the rachis is continuous.

Figure 4:E: CYK-1::GFP seems to switch from discontinuous to continuous. Is this always the case, or just due to this particular image? If it is really more continuous, what would this mean regarding the rachis bridges? Are they smaller or fused?
Response: CYK-1::GFP does not switch from discontinuous to continuous depending on *cap-1(RNAi)*. This impression was due to the two images not being taken at the exact same focal plane. CYK-1::GFP is always enriched at the rachis bridges and the discontinuity stems from the rachis openings. Depending on whether the focal plane cuts across rachis openings or not CYK-1::GFP will appear discontinuous or continuous, respectively. To avoid such confusion, we have now replaced the CYK-1::GFP images with different images where the germ cell openings are clearly visible (Figure 5E).

4H: ARX-2::GFP in controls and in the cap-1 RNAi animal, the distribution is quite varied along the GL. Is there any pattern to where ARX-2::GFP accumulates, i.e.higher at specific regions of the GL? Describing the control distribution better would help understand the change in the mutant.
Response: ARX-2::GFP has high cytoplasmic localization and no significant enrichment was observed at the rachis bridges. Although, we did observe punctate structures around the germ cells, but their localization was highly variable. We did not see any specific change in the localization pattern of the ARX-2::GFP after CAP-1 knock down, only a general increase in intensity throughout the germline (Figure 5H).

Boarder question to better understand the phenotypes: with dysregulated CP, the longer actin fibers could also be floppier, and weaker. Do any of the conditions tested suggest that loss of CAP-1 sometimes creates excess linear actin that destablilizes the structure? I don't have a good suggestion for how to image this. The discontinuous and variable rachis in cap-1 RNAi could be due to areas of excess actomyosin, next to areas of depleted actomyosin. That seems to be what Fig. 5C shows.
Response: It is entirely plausible that CP loss of function leads to longer actin fibers that are floppier and weaker. However, the longer actin fibers could be bundled by plastin and/or cross-linked by myosin II and therefore be stabilized. In any case, there currently isn't in existence any way to visualize actin in the rachis at a resolution that would answer this question. As mentioned above, the rachis is not discontinuous upon CP KD, but rather meandering. It is true that its width can be variable and this is likely due to variable contractility, similar to what we have shown for a plastin mutant in our Nat. Comm. paper.

One final suggestion: Is there evidence that could be cited that the embryos are larger, smaller or dysregulated in size when myosin and its regulators are altered? That might be a nice correlation to the observations of the shorter or taller germ cell membranes.
Response: This is an excellent suggestion. Searching the literature we found one paper where actomyosin regulators were manipulated such that NMY-2 levels at the rachis increased and embryos were smaller. This experiment, reported in Rehain-Bell et. al., 2017 https://doi.org/10.1016/j.cub.2017.01.058, involved *gck-1(RNAi)* and *ccm-3(RNAi)* and the authors reported on myosin levels at the rachis rings and on embryo length in figures 4C and 2I:

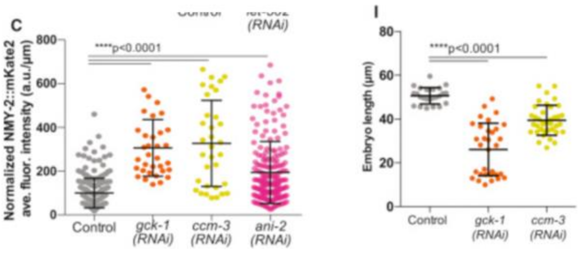

We added reference to this published result in our discussion.

© 2023. Published by The Company of Biologists under the terms of the Creative Commons Attribution License (https://creativecommons.org/licenses/by/4.0/).

Second decision letter

MS ID#: DEVELOP/2022/201099

MS TITLE: Actin capping protein regulates actomyosin contractility to maintain germline architecture in C. elegans

AUTHORS: Shinjini Ray, Priti Agarwal, Anat Nitzan, Francois Nedelec, and Ronen Zaidel-Bar

I have now received all the referees reports on the above manuscript, and have reached a decision. The referees' comments are appended below, or you can access them online: please go to BenchPress and click on the 'Manuscripts with Decisions' queue in the Author Area.

The overall evaluation is very positive and we would like to publish your manuscript in Development. Before we can proceed with formal acceptance, can you please attend to the few minor points mentioned by the reviewers.

Reviewer 2

Advance summary and potential significance to field

The authors responded to all my major concerns. I therefore find the manuscript suitable for publication in Development. A few minor issues are listed below.

Comments for the author

Minor comments on Methods section:
- Description of procedure for partial myosin RNAi and double RNAis is missing and should be added
- The response provided by the authors regarding the measurement of rachis width when the rachis is convoluted or germ cells are missing should be added
- in the fluorescence analysis, specify which background was subtracted (camera cytoplasmic?)

Reviewer 3

Advance summary and potential significance to field

Despite having been described many decades ago, the C. elegans Capping proteins, CAP-1 and CAP-2, have received limited attention. This study by Ray and colleagues directly addresses the role of CAP-1/CAPZA/Capping Protein alpha in the regulation of contractility in the C. elegans germline. The Zaidel-Bar lab recently reported a mechanistic model for the architecture of the syncytial gonad of C. elegans , and the major role played by carefully balanced actomyosin forces (Priti et al., 2018). This current study adds an important component, by analyzing the localization and role of CAP-1, which is enriched at the rachis bridge structures that are important to maintain the syncytial nature of the germline. Since they cannot use the CRISPR null mutation they generate, which arrests at the larval stage, they use RNAi throughout the paper to test the role of CAP-1, and of its regulators. The RNAi experiments are done with careful controls, and both mild and severe effects are carefully monitored. Altogether, this manuscript makes new discoveries about the role of Capping Protein in germline architecture, including that CAP-1 is required to modulate actomyosin contractility.

The authors show evidence that the germline can form without CAP-1, but the maintenance of its structures, especially the rachis, and therefore the proper formation of oocytes, requires CAP-1. Reduced CAP-1 resulted in a highly constricted rachis, with distorted oocytes, and over-expression of CAP-1 resulted also in a constricted rachis. F-actin is increased at the rachis of cap-1 RNAi animals. Since the formin CYK-1 is also enriched at the rachis bridges, they test for effects of cap-1 RNAi on CYK-1:GFP and show CYK-1::GFP can still enrich there. However, loss of cyk-1 by RNAi blocks the increased F-actin of cap-1 RNAi, suggesting it is the formins that work with CAP-1 at the rachis bridges. Loss of CAP-1 results in increased NMY-2::GFP, and phospho-MyoII, which fits with

© 2023. Published by The Company of Biologists under the terms of the Creative Commons Attribution License (https://creativecommons.org/licenses/by/4.0/).

the hyper contracted rachis phenotype. To further test if it is increased myosin/NMY-2 activity that leads to cap-1 defects, they show loss of Rho Kinase (let-502 RNAi) blocks the rachis constrictions in cap-1 RNAi, and looks like loss of let-502. FRAP and laser ablation studies further support the role of CAP-1 in regulating contractility at the rachis. Cytosim simulations to generate 128 architectures by varying parameters including the levels of actin, myosin, cross linkers and Arp2/3, then calculating the contraction rate, supported a model that the actin network is highly dense and that increased myosin would most likely increase contractility, while increased Arp2/3 would not, again supporting that the effects of CAP-1 loss are likely mediated by the effects on increased myosin.

Comments for the author

The authors have done a thorough job responding to all three Reviewers. The Reviewers comments, and the detailed response, greatly improved the presentation of the findings. Reviewing the paper once again, months later with fresh eyes, here is what I noticed:

(1). p. 4: “Interestingly, cap-1+/- deletion heterozygous mutants did not show any defect in the germline as compared to control (Figure S1E-G) suggesting that a minimum threshold reduction of the CAP-1 level is required to cause germline defects.”

This explanation may confuse readers and may be incorrect. RNAi can affect both zygotic and maternally provided gene products. The progeny of a heterozygote are maternally rescued, and therefore fine. Unless the exact amount of available protein was compared, and it is really the difference between 50% and 60% protein reduction that explains phenotypes, an alternative explanation is that maternal rescue results in the absence of phenotypes in heterozygotes. In contrast, even when RNAi does not completely eliminate the protein it can remove both maternal and embryonic gene products.

(2) p. 10 “the raise in contractility” should be “the rise….”

(3) Fig. 1C Why is NMY-2 more yellow vs. PLST-1?
Both the labeling is more yellow, and the color used in the two-color overlap, so areas of overlap look yellow, not white.

(4) Supplemental p. 10 “medium 100 more viscous than water” should that be “100 times more…”?

That is all! Nice work.

Second revision

<u>Author response to reviewers' comments</u>

Response to Reviewers - First Revision

Reviewer 2 Comments for the Author:
Minor comments on Methods section:
- Description of procedure for partial myosin RNAi and double RNAis is missing and should be added.
* We added the protocol to the materials and methods section.
- The response provided by the authors regarding the measurement of rachis width when the rachis is convoluted or germ cells are missing should be added.
* We added to the methods section this explanation: "We avoided the measurements in the regions where the germ cells were missing in different RNAi conditions and performed measurements only where clear rachis bridges were visible."
- in the fluorescence analysis, specify which background was subtracted (camera, cytoplasmic?)
* We added to the methods the sentence: "Fluorescence intensity measurement: each image was corrected for the background fluorescence present in the image outside of the worm before measurement."

© 2023. Published by The Company of Biologists under the terms of the Creative Commons Attribution License (https://creativecommons.org/licenses/by/4.0/).

Reviewer 3 Comments to author:
The authors have done a thorough job responding to all three Reviewers. The Reviewers comments, and the detailed response, greatly improved the presentation of the findings. Reviewing the paper once again, months later with fresh eyes, here is what I noticed:

(1). p. 4: “Interestingly, cap-1+/- deletion heterozygous mutants did not show any defect in the germline as compared to control (Figure S1E-G) suggesting that a minimum threshold reduction of the CAP-1 level is required to cause germline defects.”

This explanation may confuse readers and may be incorrect. RNAi can affect both zygotic and maternally provided gene products. The progeny of a heterozygote are maternally rescued, and therefore fine. Unless the exact amount of available protein was compared, and it is really the difference between 50% and 60% protein reduction that explains phenotypes, an alternative explanation is that maternal rescue results in the absence of phenotypes in heterozygotes. In contrast, even when RNAi does not completely eliminate the protein it can remove both maternal and embryonic gene products.
* We agree with the reviewer this is complicated to interpret and changed the conclusion of the experiment to: "Interestingly, cap-1+/- deletion heterozygous mutants did not show any defect in the germline as compared to control (Fig. S1E-G) suggesting that maternal CAP-1 level is sufficient for the proper germline development."

(2) p. 10 “the raise in contractility” should be “the rise….”
* We fixed this typo

(3) Fig. 1C Why is NMY-2 more yellow vs. PLST-1?
Both the labeling is more yellow, and the color used in the two-color overlap, so areas of overlap look yellow, not white.
* We changed the color scheme so in both panels it is green and magenta and colocalization gives white.

(4) Supplemental p. 10 “medium 100 more viscous than water” should that be “100 times more…”?
* We corrected this sentence as suggested.

Third decision letter

MS ID#: DEVELOP/2022/201099

MS TITLE: Actin capping protein regulates actomyosin contractility to maintain germline architecture in C. elegans

AUTHORS: Shinjini Ray, Priti Agarwal, Anat Nitzan, Francois Nedelec, and Ronen Zaidel-Bar
ARTICLE TYPE: Research Article

I am happy to tell you that your manuscript has been accepted for publication in Development, pending our standard ethics checks.

© 2023. Published by The Company of Biologists under the terms of the Creative Commons Attribution License (https://creativecommons.org/licenses/by/4.0/).
